# Comparative Chemical Profiling, Antioxidant Activity, and Antidiabetic Potential of Four Whole-Grain Red Rice Cultivars from Three Southern Border Provinces of Thailand: An In Vitro and In Silico Investigation

**DOI:** 10.3390/foods15091534

**Published:** 2026-04-28

**Authors:** Pornpen Panomwan, Pawika Mahasawat, Ittipat Meewan, Suebpong Pruttipattanapong, Nateelak Kooltheat, Thanawat Pitakpornpreecha, Sunita Makchuchit, Arunporn Itharat

**Affiliations:** 1 Faculty of Medicine, Princess of Naradhiwas University, Narathiwat 96000, Thailand; pornpen.p@pnu.ac.th; 2 Faculty of Science and Technology, Songkhla Rajabhat University, Songkhla 90000, Thailand; 3 Institute of Molecular Biosciences, Mahidol University, Nakhon Pathom 73170, Thailand; ittipat.mee@mahidol.edu (I.M.);; 4 Department of Medical Technology, School of Allied Health Sciences, Walailak University, Nakhon Si Thammarat 80160, Thailand; 5 Hematology and Transfusion Science Research Center, Walailak University, Nakhon Si Thammarat 80160, Thailand; 6 Center for Natural Rubber Latex Biotechnology Research and Innovation Development, Prince of Songkla University, Songkhla 90110, Thailand; 7 Center of Excellence in Applied Thai Traditional Medicine Research (CEATMR), Thammasat University, Pathumthani 12120, Thailand; 8 Department of Applied Thai Traditional Medicine, Faculty of Medicine, Thammasat University, Pathumthani 12120, Thailand

**Keywords:** local whole-grain red rice, Hawm Gra Dang Ngah 59, antioxidant activity, antidiabetic potential, in silico

## Abstract

**Background/Objectives**: Pigmented rice is increasingly recognized as a functional food because of its rich phytochemical composition and health-promoting potential. However, local red rice cultivars from the three southern border provinces of Thailand remain insufficiently characterized. This study comparatively evaluated four whole-grain red rice cultivars—Hawm Gra Dang Ngah 59 (HGDN 59), Hawm Mue Lau (HML), Lued Pla Lai (LPL), and Se Bu Kan Tang (SBKT)—for their chemical composition, antioxidant activities, and antidiabetic potential. **Methods**: Whole-grain rice samples were extracted with 95% ethanol and assessed for extraction yield, total phenolic content, and total flavonoid content. Antioxidant activity was measured using DPPH^•^, FRAP, and anti-lipid peroxidation assays, while antidiabetic activity was measured using α-amylase and α-glucosidase inhibition assays. LC-MS/MS-based chemical profiling, pathway classification, PCA-based chemical space analysis, molecular docking against α-glucosidase, and physicochemical/ADMET prediction were also performed. **Results**: Among the tested cultivars, HGDN 59 showed the most favorable overall profile, with the highest phenolic content, strongest antioxidant activity, and marked α-glucosidase inhibitory activity. LC-MS/MS analysis combined with docking-based screening revealed that HGDN 59 contained several abundant compounds, including ent-Epicatechin-(4α→6)-ent-epicatechin, cinnamtannin A1, apiin, and α-tocotrienol. These compounds exhibited strong binding affinities toward α-glucosidase (−10.7 to −9.6 kcal/mol), comparable to or slightly more favorable than acarbose. ADMET prediction indicated that most polyphenolic compounds exceeded Lipinski’s rule of five, while α-tocotrienol demonstrated favorable absorption property. **Conclusions**: This is the first study to suggest that HGDN 59 exhibits potential α-glucosidase inhibitory activity in vitro and may serve as a promising functional food candidate for the dietary management of postprandial glycemic response.

## 1. Introduction

The growing interest in functional foods highlights their recognized contribution to health promotion beyond basic nutritional requirements, with increasing acceptance of food as a therapeutic and preventive strategy [1]. Foods that are naturally rich in or fortified with bioactive compounds are considered functional foods because they can modulate physiological and biochemical pathways that support human health, particularly in reducing the risk of non-communicable diseases (NCDs), such as type 2 diabetes mellitus (T2DM), cardiovascular disease, and certain cancers [2]. These chronic conditions are influenced not only by nutrition and lifestyle factors but also by biological aging processes [3] and oxidative stress mechanisms [4].

Rice is one of the most widely consumed staple crops worldwide and remains a major contributor to daily dietary energy intake [5]. Increasing evidence indicates that pigmented local rice varieties in Southeast Asia are rich in phytochemicals and associated with multiple health-promoting biological activities [6,7]. In Thailand, local pigmented rice varieties such as Sangyod, Riceberry, Hom Nil, Mun Poo, and Mali Dang have attracted growing attention because of their high antioxidant capacity and diverse phytochemical composition [8,9,10,11,12,13,14]. These bioactive compounds, including phenolic acids, flavonoids, anthocyanins, and vitamin E derivatives such as tocopherols and tocotrienols, contribute significantly to antioxidant activity by scavenging reactive oxygen species (ROS) and mitigating oxidative stress. Antioxidant capacity in pigmented rice is commonly evaluated using assays such as 2,2-diphenyl-1-picrylhydrazyl radical (DPPH^•^) scavenging activity, ferric-reducing antioxidant power (FRAP) assay, and is strongly influenced by phenolic composition, with colored rice generally exhibiting higher antioxidant activity than non-pigmented varieties [15,16,17]. In addition, the anti-lipid peroxidation assay provides complementary information on antioxidant activity by reflecting the ability of the extracts to inhibit oxidative damage in lipid systems [18]. Although rice, including colored varieties, is rich in carbohydrates, it also contains diverse secondary metabolites with potential antidiabetic activities [19,20]. In particular, flavonoids from pigmented rice have been reported to be associated with the inhibition of carbohydrate-hydrolyzing enzymes such as α-amylase and α-glucosidase, which are key targets for controlling postprandial hyperglycemia [21,22,23]. Experimental studies further support the antidiabetic potential of pigmented rice. Supplementation with black, red, and brown rice extracts has been shown to reduce blood glucose levels and oxidative stress markers in diabetic models [24,25,26]. These effects are generally attributed to bioactive compounds, particularly flavonoids, which contribute to both antioxidant and enzyme-inhibitory activities [20]. In addition, pigmented rice extracts have been reported to exhibit glucose-lowering effects and may enhance insulin-signaling pathways, thereby contributing to the regulation of postprandial glycemic response [19].

Given their dual roles in antioxidant defense and modulation of carbohydrate metabolism, pigmented rice varieties have emerged as promising candidates for the dietary management of postprandial glycemic response. By delaying carbohydrate digestion and reducing glucose absorption, these bioactive compounds may help attenuate postprandial glucose excursions while simultaneously alleviating oxidative stress associated with hyperglycemia. Despite these promising findings, the relationship between phytochemical composition, antioxidant activity, and enzyme-inhibitory effects remains complex and not fully understood, particularly in locally cultivated rice varieties.

However, compared with these better-studied Thai pigmented rice cultivars, local red rice varieties cultivated in the three southern border provinces of Thailand—Yala, Pattani, and Narathiwat—remain understudied. Among these, HGDN 59 has been investigated in only a limited number of studies, including those by Yodmanee et al. [27], Panomwan and Tatiyaborworntham [28], and Chaithong et al. [29], whereas Hawm Mue Lau (HML), Lued Pla Lai (LPL), and Se Bu Kan Tang (SBKT) have yet to be scientifically reported on in terms of both chemical composition and biological properties. Therefore, this study aimed to conduct a comparative analysis of 95% ethanolic extracts from HGDN 59, HML, LPL, and SBKT with respect to extraction yield, phenolic and flavonoid contents, antioxidant activities, and antidiabetic properties, particularly α-amylase and α-glucosidase inhibitory activities. In addition, in silico molecular docking and ADMET prediction were performed to support the in vitro findings.

## 2. Materials and Methods

### 2.1. Rice Samples

**Figure 1:**
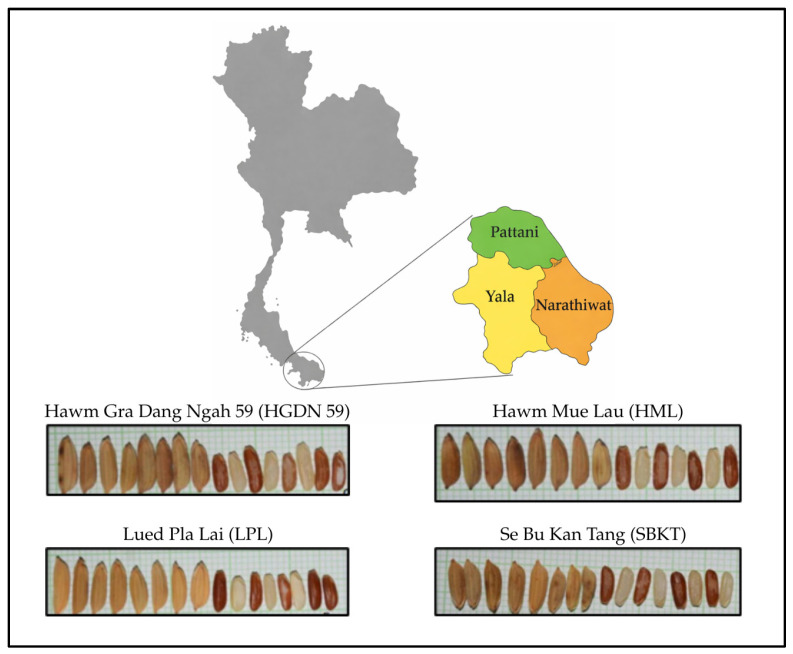
The geographical location and four whole-grain red rice varieties from the three southern border provinces of Thailand. The map was prepared by the authors with the assistance of GPT-5.3 (OpenAI), and all final editing and verification were performed by the authors.

The current study employed four Thai-grown varieties of red rice (*Oryza sativa* L.), namely Hawm Gra Dang Ngah 59 (HGDN 59), Hawm Mue Lau (HML), Lued Pla Lai (LPL), and Se Bu Kan Tang (SBKT). The geographical location of red rice varieties is shown in Figure 1. Four varieties of whole-grain rice were cultivated in the three southern border provinces of Thailand—Yala, Pattani, and Narathiwat. HGDN 59 was cultivated from early October 2021 to mid-February 2022 and harvested between late February and March 2022, whereas SBKT was cultivated from late October 2021 to mid-February 2022 and harvested between late February and March 2022. These samples were obtained from the Pattani Rice Research Center, Thailand. HML and LPL were cultivated from mid-October 2021 to early March 2022 and harvested between mid-March and April 2022. These samples were obtained from the Yala Provincial Agriculture and Cooperatives Office, Thailand.

### 2.2. Chemicals and Reagents

This study used chemicals and materials from various suppliers. These included Folin–Ciocalteu phenol reagent from Loba Chemie, India; ferrous sulfate 7-hydrate (FeSO_4_.7H_2_O) from Kemaus, Cherrybrook, NSW, Australia; ethanol and methanol from RCI Labscan, Thailand; acarbose, α-amylase, α-glucosidase enzyme, aluminum chloride (AlCl_3_), ascorbic acid, bovine brain extract type I, butylated hydroxytoluene (BHT), 2,2-diphenyl-1-picrylhydrazyl (DPPH), 3,5-dinitrosalicylic acid (DNSA), FeCl_3_, gallic acid, *p*-nitrophenyl-α-D-glucopyranoside (pNPG), potassium acetate (CH_3_CO_2_K), quercetin, sodium carbonate (Na_2_CO_3_), Thiobarbituric acid (TBA), trolox and 2,4,6-Tri- (2-pyridyl)-s-triazine (TPTZ) from Sigma-Aldrich, St. Louis, MI, USA, and phosphate- buffered saline from Vivantis, New York City, NY, USA.

### 2.3. Preparation of the Whole-Grain Red Rice Extracts

The whole-grain red rice samples were dried at 45 °C overnight and stored at room temperature. The dried samples were then ground using an electric grinder and sieved through a 100 µm test sieve (Endecotts™, Thermo Fisher Scientific, Abingdon, UK) to obtain finely ground crude samples. The samples were subsequently vacuum-packed in plastic bags and stored at −20 °C prior to extraction. The crude whole-grain rice extracts were prepared by maceration in 95% ethanol at a powder-to-solvent ratio of 1:2 (*w*/*v*), with shaking at 120 rpm for 24 h, followed by additional maceration at room temperature for 72 h. The contents were filtered through Whatman^®^ No. 1 filter paper, collected, and the residues were macerated again with 95% EtOH. This process was repeated 3 times. All filtrates were combined, and the solvents were removed by evaporation under reduced pressure at 45 °C (Hei-VAP, Heidolph, Schwabach, Germany). Subsequently, the crude extracts were dried using a freeze dryer (Gamma 2-16 LSCplus, Christ, Osterode am Harz, Germany) to obtain the dry whole-grain rice extract. The extracts were stored in a humidified cabinet until analysis took place. The extraction yield was calculated as follows:

Extraction yield (%) = (weight of dry extract/weight of powder used for extraction) × 100

### 2.4. Phytochemical Analysis

#### 2.4.1. Total Phenolic Content (TPC)

The TPC of the whole-grain rice extracts was determined following the procedure of Ainsworth and Gillespie [30] with modifications. The process involved mixing 100 µL of extract with 200 µL of 10% *v*/*v* Folin–Ciocalteu phenol reagent in distilled water, in a microcentrifuge tube with a vortex mixer (MX-S, ONilAB, San Francisco, CA, USA). Subsequently, 800 µL of 700 mM sodium carbonate was added to the mixture, which was incubated in the dark for 2 h. Gallic acid (0–50 μg/mL) served as the reference phenolic compound for the calibration curve and underwent the same treatment as the extract. The absorbance of the solution was measured at 765 nm using a UV-VIS spectrophotometer (Evolution 201, Thermo Scientific, Shanghai, China). A blank solution was prepared by substituting the extract with 100 μL of 95% ethanol and treating it identically. Data were collected from three replicates, and TPC was reported as milligrams of gallic acid equivalents (mg GAE) per gram of extract.

#### 2.4.2. Total Flavonoid Content (TFC)

The TFC of the whole-grain rice extracts was determined using the AlCl_3_ colorimetric method [31]. The process involved mixing 125 µL of extract with 375 µL of 95% ethanol, 25 µL of 10% AlCl_3_ solution, 25 µL of 1 M CH_3_CO_2_K solution, and 700 µL of distilled water. The mixture was incubated for 30 min at room temperature. Quercetin was used as a reference flavonoid; it was used to create a calibration curve over the range 0–50 μg/mL, following the same procedure. The absorbance of the solution was measured at 415 nm using a UV-VIS spectrophotometer (Evolution 201, Thermo Scientific, Shanghai, China). A blank solution was prepared by substituting the extract with 95% ethanol and treating it identically. Data were collected from three replicates, and TFC was reported as milligrams of quercetin equivalent (mg QE) per gram of extract.

### 2.5. DPPH^•^ (2,2-Diphenyl-1-Picrylhydrazyl Radical) Scavenging Activity

The antioxidant activity of the whole-grain red rice extracts was evaluated using the modified DPPH radical scavenging method from Yusoff et al. [32]. Briefly, 400 μL of whole-grain rice extracts were combined with an equal volume of freshly prepared 0.1 mM DPPH^•^ methanolic solution. The mixture was then incubated in the dark at room temperature for 30 min, after which absorbance was measured at 517 nm using a microplate reader (SPECTROstar Nano, BMG LABTECH, Ortenberg, Germany). Trolox was used as a standard antioxidant for the positive control. Three replicates were carried out, and DPPH radical scavenging activity was reported as micromoles of Trolox equivalent (µM TE) per gram of extract.

### 2.6. Ferric Reducing Antioxidant Power (FRAP) Assay

FRAP was estimated using the microplate method described by Benzie and Strain [33]. In a 96-well plate, 20 µL of the whole-grain red rice extract was mixed with 280 µL of 2,4,6-tri(2-pyridyl)-s-triazine (TPTZ) solution. The mixture was incubated at room temperature for 30 min, and the absorbance was measured at 593 nm. Ferrous sulfate (FeSO_4_) was used as the standard, and gallic acid was used as the positive control. Three replicates were carried out, and FRAP activity was expressed as ferrous equivalents (mg FeSO_4_/g of dry extract).

### 2.7. Anti-Lipid Peroxidation Activity

The inhibitory capacity of the whole-grain red rice extracts against the Fe^3+^ ascorbic acid-dependent nonenzymatic lipid peroxidation in bovine brain extract was performed according to the method of Bajpai et al. [34]. The reaction mixture was prepared from bovine brain extract type I in phosphate-buffered saline (5 mg/mL) by adding 1 mM FeCl_3_ and 1 mM ascorbic acid as a pro-oxidant in the presence of Fe, followed by incubation at 37 °C for 30 min, leading to the formation of free malondialdehyde (MDA). Different rice extract concentrations were tested for their inhibitory activity against lipid peroxidation. Thiobarbituric acid (TBA) (0.8%), butylated hydroxytoluene (BHT) (2%), and HCl (1%) were added, and the mixture was heated at 95 °C for 30 min to yield the red TBA-MDA complex. After cooling, these color products were measured colorimetrically at 532 nm using a microplate reader (SPECTROstar Nano, BMG LABTECH, Ortenberg, Germany). Three replicates were carried out, and the results were expressed as half-maximal inhibitory concentration (IC_50_), the concentration needed to inhibit lipid peroxidation by 50%. Trolox was used as the positive control.

### 2.8. Alpha-Amylase Inhibition

The inhibitory activity on α-amylase was measured using the 3,5-dinitrosalicylic acid (DNSA) method with slight modifications [35]. Briefly, 200 µL of the rice extracts were mixed with 200 µL of α-amylase enzyme solution. The mixture was incubated at room temperature for 30 min. Subsequently, 200 µL of a 1% *w*/*v* soluble starch solution was added, and the reaction mixture was incubated at room temperature for an additional 10 min. Afterward, 400 µL of color reagent was added, and the mixture was incubated at 95 °C in a heating block for 10 min. The mixture was then removed from the heating block, cooled to room temperature, and diluted with 4 mL of distilled water. The absorbance of the supernatant was measured at 540 nm using a microplate reader (SPECTROstar Nano, BMG LABTECH, Ortenberg, Germany). Three replicates were carried out, and the results were expressed as IC_50_, the concentration needed to inhibit α-amylase by 50%. Acarbose was used as the positive control.

### 2.9. Alpha-Glucosidase Inhibition

The inhibitory activity of α-glucosidase was studied using the assay of Apostolidis and Lee [35] with a slight modification. In a 96-well plate, 100 μL of whole-grain rice extracts were combined with 50 μL of α-glucosidase enzyme (1 U/mL in 0.1 M phosphate-buffered saline, pH 6.9). The mixture was incubated for 30 min at room temperature. Subsequently, 50 µL of 5 mM p-nitrophenyl-α-D-glucopyranoside (pNPG) was added as a substrate to initiate the enzymatic reaction, which was allowed to proceed for 5 min at room temperature. The α-glucosidase activity was determined by measuring absorbance at 405 nm using a microplate reader (SPECTROstar Nano, BMG LABTECH, Ortenberg, Germany). Three replicates were carried out, and the results were expressed as IC_50_, the concentration needed to inhibit α-glucosidase activity by 50%. Acarbose was used as the positive control.

### 2.10. UHPLC-ESI-QTOF-MS/MS Analysis

Chemical profiling of the whole-grain rice extracts was performed using ultra-high-performance liquid chromatography coupled with electrospray ionization quadrupole time-of-flight tandem mass spectrometry (UHPLC–ESI–QTOF–MS/MS or LC-MS/MS) (Agilent Technologies, Santa Clara, CA, USA). Chromatographic separation was achieved using a ZORBAX Eclipse Plus C18 Rapid Resolution HD column (Santa Clara, CA, USA) (150 × 2.1 mm, 1.8 µm particle size). The column temperature was maintained at 30 °C. The mobile phase consisted of solvent A (water containing 0.1% formic acid, *v*/*v*) and solvent B (acetonitrile). The flow rate was set at 0.20 mL/min, the injection volume was 2 µL, and the autosampler temperature was maintained at 25 °C. The reversed-phase chromatographic system and acidic mobile phase design were selected based on previously reported UHPLC phenolic profiling strategies for colored rice matrices, with instrument-specific optimization applied in the present study [36]. Gradient elution was performed using a multistep linear gradient from solvents A and B to enable sequential elution of polar to less polar phytochemicals, followed by column re-equilibration to initial conditions before subsequent injections, with a total analytical run time of 30 min. The UHPLC system was operated with a maximum pressure limit of 800 bar. Mass spectrometric detection was conducted using a QTOF mass spectrometer equipped with an ESI source operating in both positive and negative ion modes, acquiring full-scan MS and data-dependent MS/MS spectra over an *m*/*z* range of approximately 50–1200. Data processing was performed using Agilent MassHunter Workstation Qualitative Analysis Workflows Version B.08.00. Compound annotation was based on accurate MS/MS data, and comparison with spectral databases, including Library METLIN database, and MassHunter Personal Compound Database and Library (PCDL) Version 8.

### 2.11. In Silico Studies

#### 2.11.1. Molecular Docking

The three-dimensional structures of small molecules were obtained from the PubChem database [37]. For molecules lacking available three-dimensional structures, conformers were generated using RDKit’s experimental-torsion knowledge distance geometry (ETKDG) algorithm and energy-minimized using the Merck (Darmstadt, Germany) molecular force field (MMFF 94) [38,39]. The number of conformers generated is proportional to molecular complexity and the number of rotatable bonds counted, according to the rules previously described [40]. Gasteiger charges were computed for all structures using Open Babel [41]. The crystal structures of α-amylase (Protein data bank identifier (PDB ID): 3L2M) and α-glucosidase (PDB ID: 3L4W) were prepared by removing water molecules, existing ligands, and heteroatoms using PyMOL version 3.1.6.1 [42,43,44]. The receptors were prepared by the addition of hydrogens and assignment of partial charges from the AMBER ff99SB forcefield using the University of California, San Francisco (UCSF) Chimera DockPrep tool version 1.118 [45,46]. The substrate binding pocket of each enzyme was defined as the primary binding site. α-amylase and α-glucosidase were employed in molecular docking by Smina to screen the constructed chemical library against both enzymes [46]. Docking grids were defined with the following parameters: for alpha-amylase, center_x = 31.18, center_y = 32.36, center_z = 64.72, size_x = 39.23, size_y = 33.70, size_z = 27.71; and for alpha-glucosidase, center_x = 44.84, center_y = 85.55, center_z = 36.66, size_x = 22.80, size_y = 24.91, size_z = 23.28. The docking parameters were defined as follows: exhaustiveness = 12, maximum number of binding modes = 4, and energy range = 1 kcal/mol. The binding energy was calculated using the Vinardo scoring function [47]. The binding conformation with the lowest binding energy was chosen for protein-ligand binding analysis. The two-dimensional (2D) structures of the molecules were created using the RDKit package version 2025.09.6 in a Python version 3.12.13 environment.

#### 2.11.2. Principal Component Analysis (PCA)

To evaluate the structural diversity and distribution of the chemical components in all HGDN 59, HML, LPL, and SBKT extracts, chemical space analysis was performed. This involved clustering molecules by their molecular features into 2D space using PCA. The chemical structures of the compounds are represented by molecular fingerprints. Each obtained SMILES string was converted into an RDKit fingerprint (2048 bits) using RDKit in a Python environment [38,48]. PCA was performed using scikit-learn and visualized using the Matplotlib packages version 3.70.8 [49,50].

#### 2.11.3. Physicochemical Properties and ADMET

Relevant physicochemical properties, including molecular weight (MW), octanol-water partition coefficient (LogP), number of hydrogen bonding donors (HBD), number of hydrogen bonding acceptors (HBA), polar surface area (Å^2^) (PSA), and the structural alert for potential toxic chemical substructures, were evaluated using the SwissADME web tool (http://www.swissadme.ch/ (accessed on 3 February 2026)) [51]. These parameters were used to characterize the pharmacokinetic behavior of the candidate compounds in terms of their Absorption, Distribution, Metabolism, Excretion, and Toxicity (ADMET) profiles, providing insight into their drug-likeness and potential bioavailability. The structural information of all small molecules was represented by the simplified molecular-input line-entry system (SMILES).

### 2.12. Statistical Analysis

The experiment data were replicated at least three times and expressed as the mean ± standard deviation (SD). One-way analysis of variance (ANOVA) followed by the Bonferroni post hoc test was used to determine significant differences among samples. Correlations among assays were evaluated using Spearman’s rank correlation analysis. Half maximal inhibitory concentration (IC_50_) of each whole-grain red rice extract against α-amylase and α-glucosidase was calculated by curve fitting analysis [52]. GraphPad Prism version 10.2.3 was used for all graphs and calculations. *p* < 0.05 was considered statistically significant.

## 3. Results

### 3.1. Variations in Extraction Yield and Total Phenolic and Flavonoid Contents of Whole-Grain Red Rice Extracts

The extraction yield of pigmented rice samples ranged from 1.83 ± 0.76 in HGDN 59 to 2.99 ± 1.00% in HML, with no significant differences observed among the rice varieties. (Table 1). The variation in TPC and TFC are shown in Table 2. The TPC varied among whole-grain red rice extracts, with values ranging from 112.78 ± 3.66 mg GAE/g extract in SBKT to 190.76 ± 10.78 mg GAE/g extract in HGDN 59. The TFC also showed differences, ranging from 13.07 ± 1.98 mg QE/g extract in LPL to 32.25 ± 7.47 mg QE/g extract in SBKT. Statistical analyses indicated that the TPC and TFC of all whole-grain red rice extracts were significantly lower than those of the respective positive controls, gallic acid for TPC and quercetin for TFC (*p* < 0.001).

### 3.2. In Vitro Antioxidant Activities of Whole-Grain Red Rice Extracts

The antioxidant activities of the whole-grain red rice extracts, including DPPH radical scavenging activity, ferric reducing antioxidant power (FRAP) assay, and anti-lipid peroxidation activity, are presented in Table 3. HGDN 59 exhibited the highest antioxidant activity in terms of DPPH radical scavenging activity (897.31 ± 4.00 µM TE/g of dry extract), FRAP (158.56 ± 6.51 mg FeSO_4_/g of dry extract), and anti-lipid peroxidation activity (IC_50_ 37.87 ± 1.45 µg/mL). These values were comparable to the respective positive controls (DPPH radical scavenging activity 900.09 ± 10.61 µM TE/g of dry extract; FRAP 561.82 ± 4.37 mg FeSO_4_/g of dry extract; anti-lipid peroxidation activity (IC_50_ 14.47 ± 1.34 µg/mL)). LPL showed the least antioxidant activity in terms of DPPH radical scavenging activity and anti-lipid peroxidation activity, whereas HML showed the least FRAP.

### 3.3. In Vitro Alpha-Amylase and Alpha-Glucosidase Inhibitory Activities of Whole-Grain Red Rice Extracts

**Figure 2:**
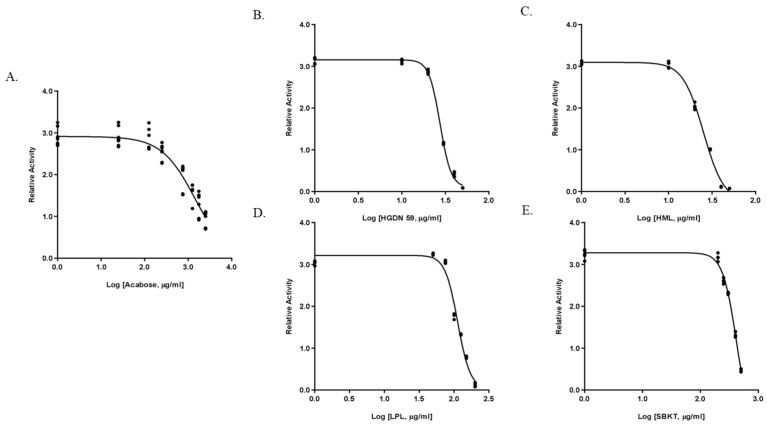
Dose–response analysis showing IC50 of α-glucosidase inhibitory activities of positive control and whole-grain red rice extracts. (**A**) Acarbose, (**B**) HGDN 59, (**C**) HML, (**D**) LPL, and (**E**) SBKT.

The IC_50_ values for α-amylase and α-glucosidase inhibition are given in Table 4. HML exhibited the most potency against α-amylase, with an IC_50_ value of 123.5 ± 0.07 µg/mL, followed by HGDN 59 (IC_50_ 356.4 ± 0.11 µg/mL). The IC_50_ value for α-amylase inhibition of the positive control, acarbose, was 86.94 ± 0.89 µg/mL. For the inhibitory action of LPL and SBKT, the IC_50_ value was 1304 ± 0.02 µg/mL and 5062 ± 0.16 µg/mL, respectively. For the IC_50_ values for α-glucosidase inhibition, HML exhibited the highest inhibitory potential (IC_50_ 25.18 ± 3.35 µg/mL), followed by HGDN 59 (27.46 ± 3.06 µg/mL). The IC_50_ values for α-glucosidase inhibition of LPL and SBKT were 112.8 ± 3.19 µg/mL and 419.6 ± 4.26 µg/mL, respectively. Interestingly, the IC_50_ values for α-glucosidase inhibition exhibited by the positive control, acarbose (IC_50_ 1484 ± 3.02 µg/mL), were higher than all rice varieties. Figure 2 shows the dose–response analysis obtained by curve fitting for determining the IC_50_ value of α-glucosidase inhibitory activity of acarbose and of each variety of whole-grain red rice extracts.

### 3.4. Chemical Profiling of Whole-Grain Red Rice Extracts

**Figure 3:**
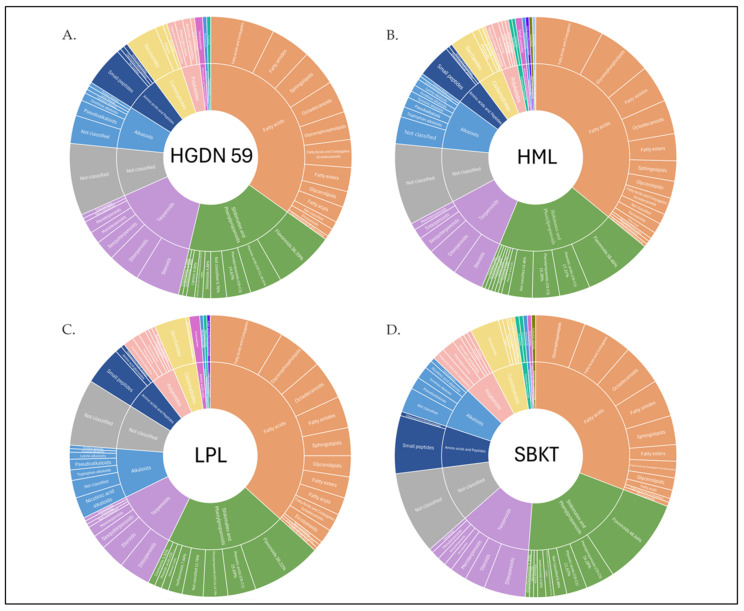
Sunburst representation of the chemical pathways and superclasses of compounds identified in whole-grain red rice extracts, using NPClassifier with percentages calculated from the frequency of annotated compounds in superclasses of shikimate and phenylpropanoid pathways. (**A**) HGDN 59, (**B**) HML, (**C**) LPL, and (**D**) SBKT. The magnified view is provided in Supplementary Figure S2.

Chemical constituents with matching scores greater than 80 and a mass accuracy within ±5 ppm were selected for further analysis, which was supported by MS/MS data and comparison with spectral databases [53,54]. LC-MS/MS analysis of whole-grain red rice extracts detected a total of 933 compounds in both negative and positive ionization modes, including 218 compounds in HGDN 59, 256 compounds in HML, 248 compounds in LPL, and 211 compounds in SBKT. These are given in Supplementary Data S1. The chemical composition of whole-grain red rice extracts by LC-MS/MS and classification by NPClassifier [55]. Chemical profiling of whole-grain red rice extracts revealed the presence of multiple pathways of compounds across all four local varieties (HGDN 59, HML, LPL, and SBKT), as shown in Figure 3. The identified compounds were classified into seven major pathways: fatty acids; shikimates and phenylpropanoids; terpenoids; alkaloids; amino acids and peptides; carbohydrates; and polyketides. In addition, several compounds were classified as pathway combinations, such as polyketides/terpenoids and shikimates and phenylpropanoids/terpenoids. Secondary metabolites associated with the shikimate and phenylpropanoid pathways represented the most abundant among the pathways identified across the four rice varieties. The distribution of shikimate and phenylpropanoid-related compounds varied among the four whole-grain red rice varieties. Flavonoids were the predominant superclass across all samples, accounting for 36.59% in HGDN 59, 38.46% in HML, 39.22% in LPL, and 48.84% in SBKT. Phenolic acids were the second major superclass, comprising 19.51% in HGDN 59, 17.31% in HML, 15.69% in LPL, and 11.63% in SBKT. Phenylpropanoids accounted for 14.63%, 15.38%, 13.73%, and 16.28% in HGDN 59, HML, LPL, and SBKT, respectively. Minor superclasses, including stilbenoids, lignans, coumarins, isoflavonoids, diarylheptanoids, phenylethanoids, and terphenyls, were also identified, although their relative abundances differed among rice varieties. In addition, compounds categorized as “not classified” accounted for 9.76% in HGDN 59, 13.46% in HML, 11.76% in LPL, and 6.98% in SBKT. Overall, SBKT showed the highest proportion of flavonoids, whereas HGDN 59 exhibited relatively higher proportions of phenolic acids compared with the other varieties.

### 3.5. PCA of the Compounds in Whole-Grain Red Rice Extracts

The chemical space analysis of the compounds found in HGDN 59, HML, LPL, and SBKT is illustrated in the PCA plot given in Supplementary Figure S3. All chemicals are mapped into two-dimensional plots of PCA based on the chemical structures represented in chemical fingerprints. According to the cluster formation across the plots, fatty acids show highly unique chemical structures, while shikimates and phenylpropanoids, terpenoids, and alkaloids are highly overlapped with other groups based on their possession of aromatic rings and conjugation systems. Carbohydrates, amino acids, and peptides are also found to form small clusters, reflecting the unique functional groups present within these compounds. The similar PCA patterns among these cultivars suggest a largely conserved chemical composition across the red rice samples.

### 3.6. Molecular Docking of the Top Candidates

**Figure 4:**
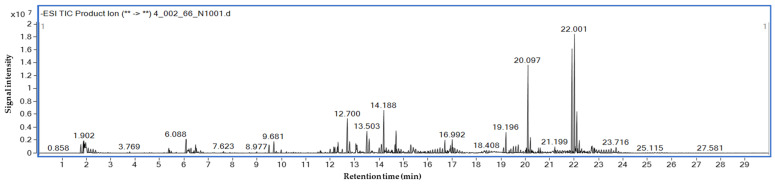
LC-MS/MS analysis of HGDN 59 in negative mode. The asterisk (**) represents ions transition.

**Figure 5:**
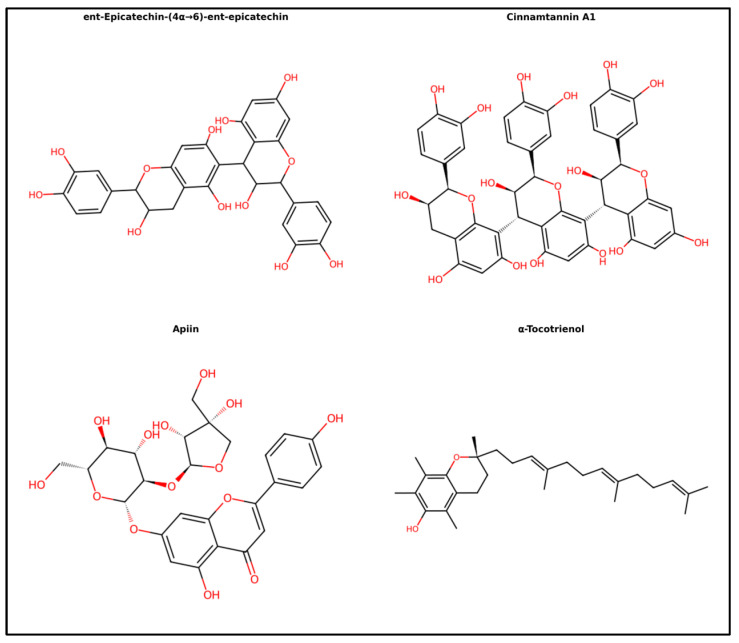
Chemical structures of the four top candidate constituents identified in HGDN 59, namely ent-Epicatechin-(4α→6)-ent-epicatechin, cinnamtannin A1, apiin, and α-tocotrienol. Carbon and oxygen atoms are colored black and red, respectively.

The top candidates among the whole-grain red rice extracts were selected based on two criteria: (i) the relative abundance of detected compounds, estimated based on the percentage peak area obtained from LC-MS/MS analysis, and (ii) their binding affinity toward α-glucosidase obtained from molecular docking. Molecular docking analysis exhibits good binding affinities ranging from −10.7 to −9.2 kcal/mol (Table 5). Notably, the HGDN 59 extract demonstrated a promising profile, exhibiting significant α-glucosidase inhibition and antioxidant activity, alongside high-abundance compounds with favorable docking scores. LC-MS/MS analysis of HGDN 59 in negative ion mode enabled the putative identification of its major constituents, as shown in Figure 4. Based on defined selection criteria, four compounds (ent-Epicatechin-(4α→6)-ent-epicatechin, cinnamtannin A1, apiin, and α-tocotrienol) (Figure 5) were predicted to be the predominant active compounds in HGDN 59 that target the α-glucosidase, with docking scores of −10.7, −10.7, −9.6, and −9.6 kcal/mol, respectively. These binding energies are comparable or slightly more favorable compared to the reference inhibitor acarbose (−9.50 kcal/mol), suggesting that the active ingredients in HGDN 59 have the potential to competitively target the active site of α-glucosidase.

**Figure 6:**
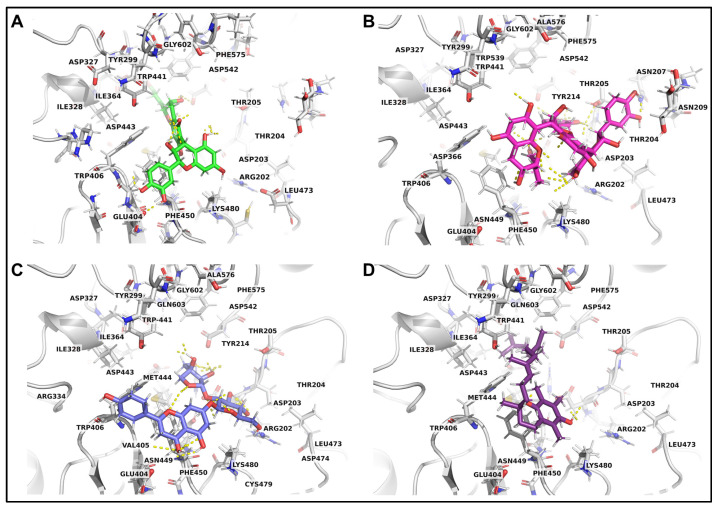
Three-dimensional binding poses of key compounds in HGDN 59 in the binding pocket of α-glucosidase. Binding conformations are shown for (**A**) ent-Epicatechin-(4α→6)-ent-epicatechin (lime), (**B**) Cinnamtannin A1 (magenta), (**C**) Apiin (slate blue), (**D**) α-tocotrienol (indigo). All ligands are displayed as sticks, and the α-glucosidase is represented as a gray stick and ribbon. Hydrogen bonds are indicated by yellow dashed lines. Oxygen, nitrogen, and sulfur atoms are colored red, blue, and gold, respectively.

**Figure 7:**
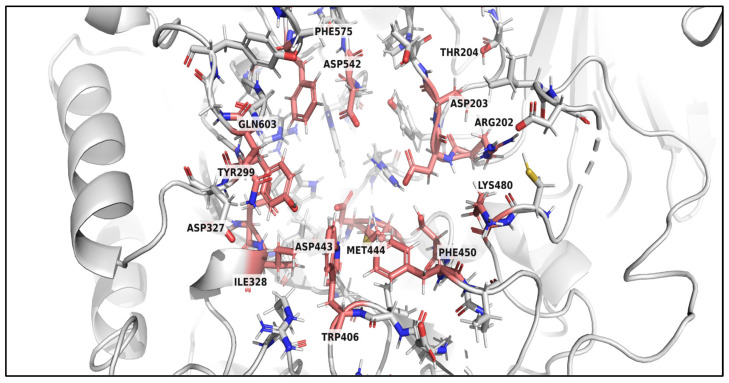
Common binding residues of α-glucosidase interacting with the compounds. The common binding residues, including ARG202, ASP203, THR204, TYR299, ASP327, ILE328, TRP406, ASP443, MET444, PHE450, LYS480, ASP542, PHE575, and GLN603, are highlighted as red sticks. The structure of α-glucosidase is represented as a gray stick and ribbon. Oxygen, nitrogen, and sulfur atoms are colored red, blue, and gold, respectively.

The binding affinities of ent-Epicatechin-(4α→6)-ent-epicatechin, cinnamtannin A1, apiin, and α-tocotrienol (Figure 6) are provided by amino acid residues TYR299, TRP406, PHE450, PHE575, ILE328, and MET444 through hydrophobic and π-stacking interactions, while THR204, and GLN603 provide polar contacts. Hydrogen-bonding interactions between ASP203, ASP327, ASP443, ASP542, and the ligands further stabilize the protein–ligand complexes. Additionally, basic residues ARG202 and LYS480 contribute to electrostatic interactions within the binding pocket. These common binding residues of α-glucosidase are depicted in Figure 7. The docking poses of other compounds identified in Table 5, that exhibit strong binding with α-glucosidase and displayed an abundant percentage peak area in other cultivars, are shown in Supplementary Figure S4.

### 3.7. Physicochemical Properties and ADMET of Four Active Compound Candidates

Four active compound candidates from HGDN 59—ent-Epicatechin-(4α→6)-ent-epicatechin, cinnamtannin A1, apiin, and α-tocotrienol—were further analyzed for their key physicochemical properties. Their absorption, distribution, metabolism, excretion, and toxicity (ADMET) were assessed to screen for favorable potency, drug-like properties, and safety. Their key physicochemical properties are shown in Table 6. Three flavonoids compounds from HGDN 59–ent-Epicatechin-(4α→6)-ent-epicatechin, cinnamtannin A1, and apiin–exceed Lipinski’s Rule of Five (Ro5) on their molecular weight (MW) (over 500 Da), number of hydrogen bonding donors (HBD) (over 5), and number of hydrogen bonding acceptors (HBA) (over 10), suggesting these compounds may be limited in their potential for intestinal absorption and permeability. While violating Ro5 suggests limited properties for conventional oral bioavailability, these flavonoids do not need to enter cells to exert their biological activities, as they would be expected to have a local inhibitory effect against α-glucosidase within the gastrointestinal tract. On the other hand, α-tocotrienol, a terpenoid compound with a MW of 424.67 Da, LogP of 2.31, HBD of 1, HBA of 2, and a low TPSA of 29.46 Å^2^, complies with all Ro5 criteria, indicating favorable absorptivity and cell permeability. These properties support its potential for intracellular uptake, which could complement the inhibitory effect of flavonoids on α-glucosidase in blocking the hydrolysis of oligosaccharides [56,57]. The compound ent-Epicatechin-(4α→6)-ent-epicatechin and cinnamtannin A1 possess one structural alert for toxicity, attributed to the presence of catechol in their structures, which are known to produce reactive oxygen species (ROS) through oxidation reaction. However, this potential is mitigated by the strong antioxidant capacity demonstrated by HGDN 59 in the DPPH radical scavenging assay, suggesting that the abundance of antioxidative components in HGDN 59 is capable of neutralizing the produced ROS through the oxidation of catechol. Additionally, apiin and α-tocotrienol presented no toxic substructure. This suggests a low toxicity profile across these four compounds. Additionally, the physicochemical properties of other compounds in HGDN 59 that were not identified as potential ingredient candidates, as well as the compounds HML, LPL, and SBKT cultivars that did not pass the selection criteria, are provided in Supplementary Table S1.

## 4. Discussion

This study presents the first comprehensive investigation of four local red rice varieties—Hawm Gra Dang Ngah 59 (HGDN 59), Hawm Mue Lau (HML), Lued Pla Lai (LPL), and Se Bu Kan Tang (SBKT)—cultivated in the three southern border provinces of Thailand (Yala, Pattani, and Narathiwat). Growing evidence indicates that traditional pigmented rice varieties in Southeast Asia are rich in phytochemicals and associated with diverse health-promoting biological activities [6]; however, scientific data describing the phytochemical composition and bioactivities of these specific local red rice varieties remain limited. Therefore, the present study aimed to analyze the chemical profiles and evaluate antioxidant and antidiabetic properties relevant to the prevention of NCDs. In addition, in silico molecular docking and physicochemical property prediction for evaluating their absorption, distribution, metabolism, excretion, and toxicity (ADMET) were provided to support the in vitro findings.

The variation in extraction yield and phytochemical contents observed among the four varieties reflects both intrinsic varietal differences and solvent-matrix interactions during extraction. In the study, ethanol extraction was selected because it is considered safe for food and nutraceutical applications, making the resulting extracts more relevant for dietary and functional ingredient development [58,59,60,61]. Moreover, ethanol has intermediate polarity and is suitable for extracting a broad range of bioactive constituents, particularly phenolic acids and flavonoids, which are major components of the functional properties of pigmented rice [20,29]. This is consistent with the phytochemical profiles and biological activities observed in the present study, and supports the suitability of ethanol extraction for screening compounds in these four whole-grain red rice extracts.

Comparison among the four varieties showed that HML and SBKT produced relatively higher extraction yields, whereas HGDN 59 exhibited the highest total phenolic content, followed by LPL. This indicates that extraction yield does not necessarily correspond to phenolic content, but rather reflects the total amount of extractable material, including carbohydrates, proteins, and other non-phenolic constituents. Similar solvent-dependent discrepancies between extraction yield and phenolic recovery have been reported, in which conditions giving the highest yield did not produce the highest TPC [62,63]. The extraction yields obtained in the present study (1.83–2.99%) were higher than those reported in some ethanol-based rice extraction studies of roasted broken brown rice powder, where crude extract recovery was below 1% of the starting material [64], likely due to differences in raw material characteristics and extraction conditions.

For total phenolic content, ranging from 112.78 to 190.76 mg GAE/g extract, and total flavonoid content ranging from 13.07 to 32.25 mg QE/g extract, the values observed in the present study are consistent with previously reported broader ranges from Thai pigmented rice, with TPC values of approximately 18–189 mg GAE/g rice extract and flavonoid contents of 19–46 mg rutin eq/g rice extract [65], and in some pigmented varieties, up to 128–322 mg GAE/g extract and 86–341 mg CE/g extract [66]. Direct numerical comparison across studies should be interpreted with caution, because flavonoid contents expressed as QE, rutin eq, or CE are not directly interchangeable, and because results are strongly influenced by solvent composition, extraction technique, particle size, and grain matrix characteristics, which affect solvent penetration and solute solubility [67].

Antioxidant activities were evaluated using DPPH radical scavenging activity, FRAP assay, and anti-lipid peroxidation activity. The results showed marked differences among the four whole-grain red rice extracts, indicating variation in their bioactive potential and antioxidant mechanisms. Different positive controls were used for each assay according to standard protocols, as each method is based on different reaction mechanisms. Trolox was used for the DPPH radical scavenging activity, and anti-lipid peroxidation activity, while gallic acid was used for the FRAP assay. These assays were not intended for direct comparison, but rather to provide complementary evaluation of antioxidant activity using method-specific standards. HGDN 59 exhibited the strongest antioxidant activities across the DPPH radical scavenging activity, FRAP assay, and anti-lipid peroxidation activity, with DPPH radical scavenging activity approaching that of the Trolox positive control, suggesting a high radical-quenching capacity. Similar trends have been reported in pigmented rice studies, where ethanolic extracts demonstrated strong antioxidant activity and where DPPH^•^ and FRAP values varied depending on cultivar and extraction conditions [6]. Previous studies on Thai and Asian pigmented rice varieties, including Riceberry, Sangyod, and black rice, have shown that colored rice generally possesses higher antioxidant capacity than non-pigmented rice due to enriched phytochemical composition [20]. The evidence from previous studies supports the use of ethanol for its ability to extract a broad range of bioactive compounds, including phenolics and flavonoids, and to enhance the recovery of polyphenols associated with antioxidant activity [29,63,68,69]. Likewise, studies on Hawm Gra Dang Ngah rice (HGDN) have demonstrated strong antioxidant capacity in ethanolic extracts, consistent with the high activity observed for HGDN 59 in the present study [29]. In contrast, the FRAP values of all rice extracts were considerably lower than those of the gallic acid control. This is expected because crude extracts contain complex mixtures of compounds with varying reducing abilities compared with pure phenolic standards. Likewise, anti-lipid peroxidation activity showed higher IC_50_ values than Trolox, indicating lower potency but still demonstrating measurable protection against lipid oxidation.

Notably, SBKT exhibited relatively strong antioxidant capacity despite not having the highest total phenolic content, whereas LPL showed weaker DPPH radical scavenging and anti-lipid peroxidation activities, and HML demonstrated lower reducing power in the FRAP. These findings suggest that antioxidant activity is influenced not only by total phenolic concentration, but also by the composition, structural characteristics, and potential synergistic interactions among individual phytochemicals [70,71,72]. The antioxidant patterns observed were generally supported by the phytochemical data, with HGDN 59 showing the highest total phenolic content, consistent with previous reports identifying phenolic compounds as major contributors to antioxidant capacity through electron transfer and radical-scavenging mechanisms [73]. However, total flavonoid content did not directly correlate with antioxidant strength, indicating that qualitative differences in phenolic composition may play a more important role than total flavonoid quantity alone [74,75]. Collectively, these findings demonstrate that antioxidant activity among local red rice varieties is determined by both phenolic abundance and varietal differences in phytochemical profiles, supporting the potential of local red rice varieties as functional food sources rich in antioxidant-active compounds, especially HGDN 59.

In vitro α-amylase and α-glucosidase inhibitory activities observed in whole-grain red rice extracts suggest their potential role in modulating carbohydrate digestion and postprandial glucose responses. As these enzymes are responsible for starch hydrolysis into absorbable sugars, their inhibition represents an effective strategy for controlling postprandial hyperglycemia and managing type 2 diabetes [76,77,78]. Among the tested samples, HML and HGDN 59 showed relatively strong inhibitory activity against α-glucosidase, with IC_50_ values markedly lower than those observed for α-amylase, whereas LPL and SBKT exhibited weaker inhibitory effects. Supporting evidence from analysis of methanol extracts of *Jumli Marshi* rice from Nepal indicates weak α-amylase (EC_50_ >1000 μg/mL) inhibition compared to α- glucosidase with EC_50_ 250 ± 2.5 µg/mL [79]. This pattern suggests that the rice extracts may exert more selective inhibition toward α-glucosidase rather than α-amylase. Such selective inhibition is considered beneficial in dietary strategies for glycemic control, as strong α-glucosidase inhibition combined with moderate α-amylase inhibition may reduce postprandial glucose spikes while minimizing gastrointestinal side effects commonly associated with excessive α-amylase inhibition [80].

The inhibitory effects observed in the present study are likely associated with the interaction between phenolic compounds and digestive enzymes. Previous studies have demonstrated that phenolics can inhibit α-amylase and α-glucosidase through non-covalent interactions, including hydrogen bonding between hydroxyl groups and catalytic residues, as well as hydrophobic interactions involving aromatic rings [21,22,23]. Structural characteristics of phenolic compounds play a crucial role in determining inhibitory strength; for example, hydroxycinnamic acids such as caffeic, ferulic, *p*-coumaric, and sinapic acids containing conjugated double bonds and carbonyl groups exhibit enhanced binding stability within enzyme active sites of α-amylase, while flavonoids with catechol structures and, hydroxyl substitutions, and phenolic acids —such as methyl vanillate, syringic acid, and vanillic acid from Thai colored rice— have been reported as potent α-glucosidase inhibitors [22,23,81]. These mechanisms may explain the stronger α-glucosidase inhibition observed in HML and HGDN 59 extracts, where HGDN 59 showed the highest antioxidant activity, and HML demonstrated notable lipid peroxidation inhibitory activity compared with LPL and SBKT, potentially due to the diverse flavonoid-related compounds identified by LC-MS/MS analysis.

In addition to direct enzyme inhibition, polyphenols (such as phenolic acids, flavonoids, tannins, stilbenes, curcuminoids, lignans, and others) may influence starch digestion by interacting with starch molecules, thereby limiting enzyme accessibility and slowing glucose release [82]. This mechanism provides a plausible explanation for the relatively weaker α-amylase inhibition observed in the present study. Starch–polyphenol inclusion complexes (corn, wheat, and rice) depend on their structure and the characteristics of the food matrix. These starch–polyphenol interactions may lead to the formation of ordered crystalline or lamellar structures that reduce starch susceptibility to enzymatic hydrolysis, thereby lowering glucose release without requiring strong direct inhibition of α-amylase [82]. Similar reductions in starch digestibility have been reported for phenolic-rich extracts from natural sources such as *Lonicera caerulea* berry, where the effects were attributed to both enzyme inhibition and polyphenol–starch interactions [83]. Furthermore, these complexes may reduce enzyme access to starch substrates while simultaneously modulating polyphenol bioaccessibility, suggesting that matrix effects play an important role in digestion behavior. Therefore, the potential for α-amylase and α-glucosidase inhibitory activity observed in whole-grain red rice extracts likely results from combined mechanisms involving selective enzyme inhibition and starch–polyphenol interactions that collectively slow starch digestion. Further studies investigating inhibition kinetics and structural characterization of starch–polyphenol complexes would help clarify the precise mechanisms underlying the modulation of α-amylase activity.

Spearman’s rank correlation analysis of phytochemical contents and biological activities was performed (data shown in Supplementary Figure S1). A strong positive correlation between DPPH^•^ and FRAP (ρ = 0.80) indicates good agreement between the antioxidant assays. Moderate positive correlations between anti-lipid peroxidation activity (ATLP) and enzyme-inhibitory activities (α-amylase and α-glucosidase; IAA and IAG, ρ ≈ 0.60), as well as between FRAP and α-glucosidase inhibition (ρ ≈ 0.40), suggest that redox-related mechanisms may partially contribute to enzyme inhibition. In contrast, TPC and TFC exhibited weak to moderate correlations with most bioactivities, indicating that overall phenolic abundance alone may not fully explain the observed effects. This implies that specific bioactive constituents, rather than bulk phenolic levels, are likely responsible, consistent with the LC–MS/MS-based identification and molecular docking results. Nevertheless, due to the limited sample size, these correlations should be interpreted with caution, and further studies with larger datasets are required to confirm these relationships.

Compound annotation of ethanolic HGDN 59, HML, SBKT, and LPL extracts was performed using LC-MS/MS analysis, as summarized in Supplementary Data S1. The identification was based on accurate mass measurements, characteristic MS/MS fragmentation patterns, and spectral matching against databases, including METLIN and the Personal Compound Database and Library (PCDL), with a mass tolerance of ±5 ppm and matching scores above 80. Based on these criteria, the detected compounds were classified as putatively annotated (Level 2) according to the Metabolomics Standards Initiative [84,85]. This level of confidence is considered sufficient for subsequent bioactivity-guided analysis, including the selection of candidate compounds for molecular docking and the interpretation of their potential α-glucosidase inhibitory activity. The shikimate and phenylpropanoid pathways serve as key biosynthetic routes leading to diverse classes of polyphenolic compounds (PCs), such as flavonoids, phenolic acids, isoflavonoids, lignans, stilbenoids, coumarins, and other phenylpropanoid derivatives, which compose the largest groups of PCs [86]. Moreover, the rice grain coloration is mainly due to the accumulation of flavonoids [20].

The PCA-based chemical space analysis provided additional insight into the structural diversity of compounds identified in ethanolic whole-grain red rice extracts and supported the pathway distribution observed in the sunburst classification profiles. To the best of our knowledge, this study represents the first PCA-based chemical space analysis of local red rice cultivars from the three southern border provinces of Thailand. Although the four rice varieties showed broadly similar clustering patterns, differences in compound distribution across the PCA space reflected varietal variation in chemical composition. Fatty acid-related compounds formed clearly separated clusters, indicating distinct structural features associated with long aliphatic chains and lower aromaticity, which is consistent with their dominant representation in the sunburst pathway analysis. In contrast, compounds associated with the shikimate and phenylpropanoid pathways —including flavonoids, phenolic acids, and related secondary metabolites—showed substantial overlap, suggesting shared structural characteristics such as aromatic rings and conjugated systems. Similar observations have been reported in chemical-space studies based on molecular fingerprints, where structurally related natural products tend to cluster together despite chemical diversity within superclasses [87]. The compact clustering of carbohydrates and amino acids/peptides further supports their relatively conserved structural features compared with secondary metabolites. These findings highlight that ethanol extraction, widely recognized for efficiently recovering structurally diverse phenolic and semi-polar compounds from rice matrices, enables broad coverage of chemical classes and contributes to the observed diversity in PCA space [20,29]. Overall, the PCA complements the sunburst classification by demonstrating that primary metabolites occupy distinct chemical regions. In contrast, secondary metabolites derived from shikimate and phenylpropanoid pathways form overlapping yet chemically diverse clusters, reflecting both shared biosynthetic origins and structural variability among local red rice varieties.

This is the first study to combine chemical profiling, molecular docking, and physicochemical prediction to identify HGDN 59 as a local red rice variety exhibiting potential α-glucosidase inhibitory activity in vitro, with potential for functional food development. According to LC-MS/MS analysis combined with docking-based screening, HGDN 59 contained several highly abundant chemical compounds, including ent-Epicatechin-(4α→6)-ent-epicatechin (9.04%), α-tocotrienol (7.42%), apiin (3.50%), and cinnamtannin A1 (2.11%), with binding affinities toward α-glucosidase of −10.7 to −9.6 kcal/mol, slightly more favorable than and comparable to the reference inhibitor acarbose (−9.5 kcal/mol). Regarding the study by Chaithong et al. [29], ethanolic HGDN extracts showed significantly stronger α-glucosidase inhibition than their water extract, attributed to higher phenolic, flavonoid, and anthocyanin content. These findings suggest that the strong α-glucosidase inhibitory potential observed for HGDN 59 may be attributed to the high abundance of flavonoid-related compounds identified by LC-MS/MS, particularly ent-Epicatechin-(4α→6)-ent-epicatechin, cinnamtannin A1, apiin, and α-tocotrienol. Molecular docking demonstrated that these compounds exhibited high binding affinities toward α-glucosidase, comparable to or stronger than acarbose, supporting their potential for enzyme modulation. The inhibitory behavior is consistent with previous studies reporting that hydroxylated flavonoids such as naringenin, epicatechin, and epigallocatechin achieve stable binding within α-glucosidase through hydrogen bonding and hydrophobic interactions, particularly involving hydroxyl and ketonic functional groups that enhance binding stability and ligand efficiency [88,89]. Moreover, previous studies have demonstrated that flavonoid subclasses differ in their α-glucosidase inhibitory potency, with anthocyanidins generally showing stronger inhibition than isoflavones, flavonols, flavones, flavanones, and flavan-3-ols, respectively [56]. This structure-dependent behavior supports our findings and suggests that differences in flavonoid composition among the four red rice cultivars may have contributed, at least in part, to the variation in α-glucosidase inhibitory activity observed in the present study.

Similar interaction patterns were observed in the present study, in which the top HGDN 59 compounds formed multiple stabilizing contacts within the catalytic pocket, highlighting the key structural features in determining inhibitory potency, such as aromatic rings and hydroxyl substitutions. Specifically, docking analysis revealed that these compounds interacted with critical α-glucosidase residues, including ASP203, TYR299, ASP327, TRP406, and ASP443 through hydrogen bonding, π-stacking, and hydrophobic interactions for ligand–enzyme complex stabilization. These residues align with the known binding site of α-glucosidase for acarbose, with the docking pose illustrated in Supplementary Figure S5, highlighting an inhibitory profile similar to acarbose. Moreover, these interaction patterns are consistent with recent mechanistic studies demonstrating that flavonoids and procyanidin derivatives inhibit carbohydrate-hydrolyzing enzymes via multipoint interactions at catalytic and peripheral binding sites, thereby slowing carbohydrate digestion and postprandial glucose release [80]. Although direct kinetic data for the four identified active compounds have yet to be reported, their structural similarity to known flavonoid-based inhibitors suggests potential for α-glucosidase inhibitory activity and possible effects on carbohydrate digestion and postprandial glucose regulation. However, detailed mechanistic studies and in vivo evaluations remain necessary to validate the proposed effects. Notably, although cinnamtannin A1 was also detected in SBKT, the higher peak abundance and broader diversity of compounds in HGDN 59 likely explain its stronger overall activity, highlighting the importance of chemical composition rather than the presence of individual compounds alone, consistent with the LC–MS/MS-based identification and molecular docking results.

In addition to docking performance, ADMET prediction based on physicochemical property evaluation supported the functional food potential of HGDN 59 compounds. Physicochemical properties of ent-Epicatechin-(4α→6)-ent-epicatechin, cinnamtannin A1, and apiin exceeded the Ro5 thresholds, primarily attributable to their large and highly hydroxylated polyphenolic structures. However, such behavior is common for natural polyphenols and does not necessarily preclude biological efficacy when consumed as food matrices rather than isolated drugs [82]. Despite their limitation with regard to intestinal absorptivity and permeability, these compounds are proposed to exert their antidiabetic effects through local α-glucosidase activity in the gastrointestinal tract, without requiring cellular uptake. In contrast, α-tocotrienol was fully compliant with Ro5, demonstrating its favorable absorptivity and permeability. This property may additionally confer intracellular inhibitory activity of α-tocotrienol for the glycogen degradation function of α-glucosidase. Collectively, the abundance of these bioactive compounds in HGDN 59 suggests that regular consumption of this cultivar may be beneficial in reducing postprandial glucose excursions through α-glucosidase inhibition in the gastrointestinal tract and intracellular mechanisms. This interpretation is further supported by a previous report identifying HGDN as a low-glycemic-index rice variety [90], which may also contribute to lower postprandial glucose and insulin responses.

## 5. Conclusions

The present study investigated four local red rice cultivars—HGDN 59, HML, LPL, and SBKT—cultivated in the three southern border provinces of Thailand (Yala, Pattani, and Narathiwat). Whole-grain rice extracts obtained using 95% ethanol were evaluated for extraction yield, total phenolic and flavonoid contents, antioxidant activities, and α-amylase and α-glucosidase inhibitory activities, together with LC-MS/MS-based chemical profiling. Among the tested cultivars, HGDN 59 exhibited the highest antioxidant capacity and notable α-glucosidase inhibitory activity under in vitro conditions. Chemical profiling combined with PCA enabled the characterization of major constituents in HGDN 59 extracts. Based on defined selection criteria, four compounds —ent-Epicatechin-(4α→6)-ent-epicatechin, cinnamtannin A1, apiin, and α-tocotrienol—were proposed as major contributors, supported by molecular docking and ADMET prediction, which indicated favorable interactions with α-glucosidase. Collectively, these findings suggest that HGDN 59 exhibits potential α-glucosidase inhibitory activity in vitro and may serve as a promising functional food candidate for the dietary management of postprandial glycemic response. However, further studies, including cell-based assays, in vivo experiments, and clinical investigations, are required to validate these effects and clarify the underlying mechanisms.

## Figures and Tables

**Table 1:** The extraction yield (%) of whole-grain red rice extracts.

Rice Varieties	Extraction Yield (%)
HGDN 59	1.83 ± 0.76
HML	2.99 ± 1.00
LPL	2.45 ± 1.74
SBKT	2.63 ± 0.79

**Table 2:** Total phenolic and flavonoid contents of whole-grain red rice extracts.

Rice Varieties	TPC (mg GAE/g Extract)	TFC (mg QE/g Extract)
HGDN 59	190.76 ± 10.78 *	13.52 ± 2.36 *
HML	127.49 ± 1.54 *	25.31 ± 5.07 *
LPL	138.50 ± 12.48 *	13.07 ± 1.98 *
SBKT	112.78 ± 3.66 *	32.25 ± 7.47 *

Note: Values are expressed as mean ± SD, *n*  =  3. One-way ANOVA, compared with positive control: * *p* < 0.001.

**Table 3:** Antioxidant activity of the whole-grain red rice extracts.

Rice Varieties/ Positive Controls	DPPH^•^ Scavenging Activity (µM TE/g of Dry Extract)	FRAP Assay (mg FeSO_4_/g of Dry Extract)	Anti-Lipid Peroxidation Activity, IC_50_ (µg/mL)
HGDN 59	897.31 ± 4.00 *	158.56 ± 6.51 *	37.87 ± 1.45
HML	422.31 ± 40.78 *	25.53 ± 1.56 *	62.74 ± 0.31 ^a^
LPL	391.30 ± 13.91 *	58.45 ± 1.52 *	229.03 ± 22.18 *
SBKT	825.09 ± 30.18 *	61.30 ± 8.45 *	89.69 ± 2.50 *
Positive controls	900.09 ± 10.61 (Trolox)	561.82 ± 4.37 (Gallic acid)	14.47 ± 1.34 * (Trolox)

Note: Values are expressed as mean ± SD, *n*  =  3. One-way ANOVA, compared with positive control: * *p* < 0.001 and ^a^ *p* = 0.0006.

**Table 4:** IC50 of α-amylase and α-glucosidase inhibitory activities of whole-grain red rice extracts.

Rice Varieties/Positive Control	α-Amylase IC_50_ (µg/mL)	α-Glucosidase IC_50_ (µg/mL)
HGDN 59	356.4 ± 0.11 *	27.46 ± 3.06 *
HML	123.5 ± 0.07 *	25.18 ± 3.35 *
LPL	1304 ± 0.02 *	112.8 ± 3.19 *
SBKT	5062 ± 0.16 *	419.6 ± 4.26 *
Acarbose	86.94 ± 0.89	1484 ± 3.02

Note: Values are expressed as mean ± SD, *n*  =  3. One-way ANOVA, compared with positive control: * *p* < 0.001.

**Table 5:** The docking scores of compounds from whole-grain red rice extracts in the binding site of the α-glucosidase target.

No.	RT (min)	Name of Compound	Molecular Formula	Pubchem CID	*m*/*z*	Peak Area (%)	Docking Score (kcal/mol)	Superclass
**HGDN 59 (Negative mode)**								
1.	6.14	ent-Epicatechin-(4α→6)-ent-epicatechin	C_30_H_26_O_12_	13990892	577.1355	9.04	−10.7	Flavonoids
2.	6.265	Cinnamtannin A1	C_45_H_38_O_18_	169853	865.1979	2.11	−10.7	Flavonoids
3.	6.792	Procyanidin B2	C_30_H_26_O_12_	122738	579.1494	0.02	−10.2	Flavonoids
4.	7.693	Natsudaidain 3-(4-O-3-hydroxy-3 methylglutaroylglucoside)	C_33_H_40_O_18_	73092145	747.2106	0.02	−10.1	Flavonoids
5.	6.641	Apiin	C_26_H_28_O_14_	5280746	563.1406	3.5	−9.6	Flavonoids
6.	12.08	α-tocotrienol	C_29_H_44_O_2_	5282347	423.3262	7.42	−9.6	Terpenoids
**HML (Negative mode)**								
7.	9.876	Rutin	C_27_H_30_O_16_	5280805	669.1676	1.15	−9.5	Flavonoids
**HML (Positive mode)**								
8.	7.353	Isorhamnetin 3-O-[b-L-rhamnofuranosyl-(1->6)-D-glucopyranoside]	C_28_H_32_O_16_	21159149	642.2009	0.1	−10.7	Flavonoids
9.	7.82	Peonidin 3-galactoside-5-glucoside	C_28_H_33_O_16_	44256835	625.1741	0.09	−9.8	Flavonoids
**LPL (Negative mode)**								
10.	7.553	Quercetin 3,3’-dimethyl ether 7-rutinoside	C_29_H_34_O_16_	44259683	637.1764	0.01	−9.7	Flavonoids
**LPL (Positive mode)**								
11.	11.047	Tyr Trp Gly	C_22_H_24_N_4_O_5_	145458785	425.1839	0.08	−11.4	Small Peptides
**SBKT (Negative mode)**								
12.	6.261	Cinnamtannin A1	C_45_H_38_O_18_	169853	865.1979	2.03	−10.7	Flavonoids
**SBKT (Positive mode)**								
13.	2.056	Hexandraside E	C_32_H_38_O_16_	44258787	362.0976	4.82	−9.5	Flavonoids
14.	6.013	Asp Trp Glu	C_20_H_24_N_4_O_8_	145454580	471.1467	0.01	−10.4	Small peptides
15.	12.425	Asn Tyr Lys	C_19_H_29_N_5_O_6_	145454304	441.246	0.12	−9.2	Small peptides

**Table 6:** Physicochemical properties of four compounds of HGDN 59 extracts identified as active ingredients targeting α-glucosidase, including molecular formular, MW, LogP, HBD, HBA, TPSA, and structural alert.

No.	Name of Compound	Molecular Formula	MW	LogP ^a^	HBD ^b^	HBA ^c^	TPSA ^d^	Structural Alert ^e^
1.	ent-Epicatechin-(4α→6)-epicatechin	C_30_H_26_O_12_	578.526	1.80	10	12	220.76	1
2.	Cinnamtannin A1	C_45_H_38_O_18_	866.781	2.50	15	18	331.14	1
3.	Apiin	C_26_H_28_O_14_	564.496	2.31	8	14	228.97	0
4.	α-tocotrienol	C_29_H_44_O_2_	424.669	2.31	1	2	29.46	0

^a^ LogP values represent the octanol–water partition coefficient. ^b^ HBD represents the number of hydrogen bonding donors. ^c^ HBA represents the number of hydrogen bonding acceptors. ^d^ TPSA represents the polar surface area (Å^2^). ^e^ Structural alert count reflects the number of toxicophoric substructures identified within each compound, with higher values indicating a greater likelihood of toxicity.

## Data Availability

The data presented in this study are available in the article and Supplementary Materials. Further inquiries can be directed to the corresponding author.

## Supplementary Materials

The following supporting information can be downloaded at: https://www.mdpi.com/article/10.3390/foods15091534/s1, Data S1: Chemical profiling (positive and negative modes) of four whole-grain red rice extracts: HGDN 59, HML, LPL and SBKT; Figure S1: Spearman’s rank correlation analysis of phytochemical contents and biological activities; Figure S2: The magnified view of chemical pathways and superclasses of compounds identified in whole-grain red rice extracts; Figure S3: The PCA plots of the chemical space of the compositions across HGDN 59, HML, LPL, and SBKT cultivars.; Figure S4: Three-dimensional binding poses of compounds identified from HGDN 59 (negative mode), HML (negative and positive modes), LPL (negative and positive modes), and SBKT (negative and positive modes) cultivars in the binding pocket of α-glucosidase; Figure S5: Three-dimensional binding pose of acarbose, a known inhibitor of α-glucosidase, within the enzyme’s active site; Table S1: Physicochemical properties of other compounds identified in other cultivar extracts targeting α-glucosidase, including molecular formular, MW, LogP, HBD, HBA, TPSA, and structural alert.

## Abbreviations

The following abbreviations are used in this manuscript:

ADMET	Absorption, distribution, metabolism, excretion, and toxicity
ANOVA	One-way analysis of variance
ATLP	Anti-lipid peroxidation activity
CE	Catechin equivalents
DNSA	3,5-dinitrosalicylic acid
DPPH	2,2-diphenyl-1-picrylhydrazyl
ETKDG	Experimental-torsion knowledge distance geometry
FRAP	Ferric reducing antioxidant power
GAE	Gallic acid
HBA	Hydrogen-bonding acceptors
HBD	Hydrogen-bonding donors
HGDN 59	Hawm Gra Dang Ngah 59
HML	Hawm Mue Lau
IAA	Inhibitory activity of α-amylase
IAG	Inhibitory activity of α-glucosidase
LC–MS/MS	Liquid chromatography coupled with tandem mass spectrometry
LPL	Lued Pla Lai
MDA	Malondialdehyde
MMFF 94	Merck molecular force field 94
NCDs	Non-communicable diseases
PCA	Principal Component Analysis
PCDL	Personal compound database and library
PCs	Polyphenolic compounds
PDB ID	Protein data bank identifier
*p*NPG	*p*-nitrophenyl-α-D-glucopyranoside
QE	Quercetin
SBKT	Se Bu Kan Tang
SD	Standard deviation
SMILES	Simplified molecular-input line-entry system
T2DM	Type 2 diabetes mellitus
TBA	Thiobarbituric acid
TE	Trolox
TFC	Total flavonoid content
TPC	Total phenolic content
2D	Two dimensional
UCSF	University of California, San Francisco
