# Supplementary material for: Comparative Chemical Profiling, Antioxidant Activity, and Antidiabetic Potential of Four Whole-Grain Red Rice Cultivars from Three Southern Border Provinces of Thailand: An In Vitro and In Silico Investigation
Source: Foods. 2026 Apr 28;15(9):1534. doi: 10.3390/foods15091534 (PMC13164330; doi:10.3390/foods15091534)
Supplement: Supplementary file 1 [file foods-15-01534-s001.zip › Figure S1, S3, S4, S5 and Table S1.pdf]

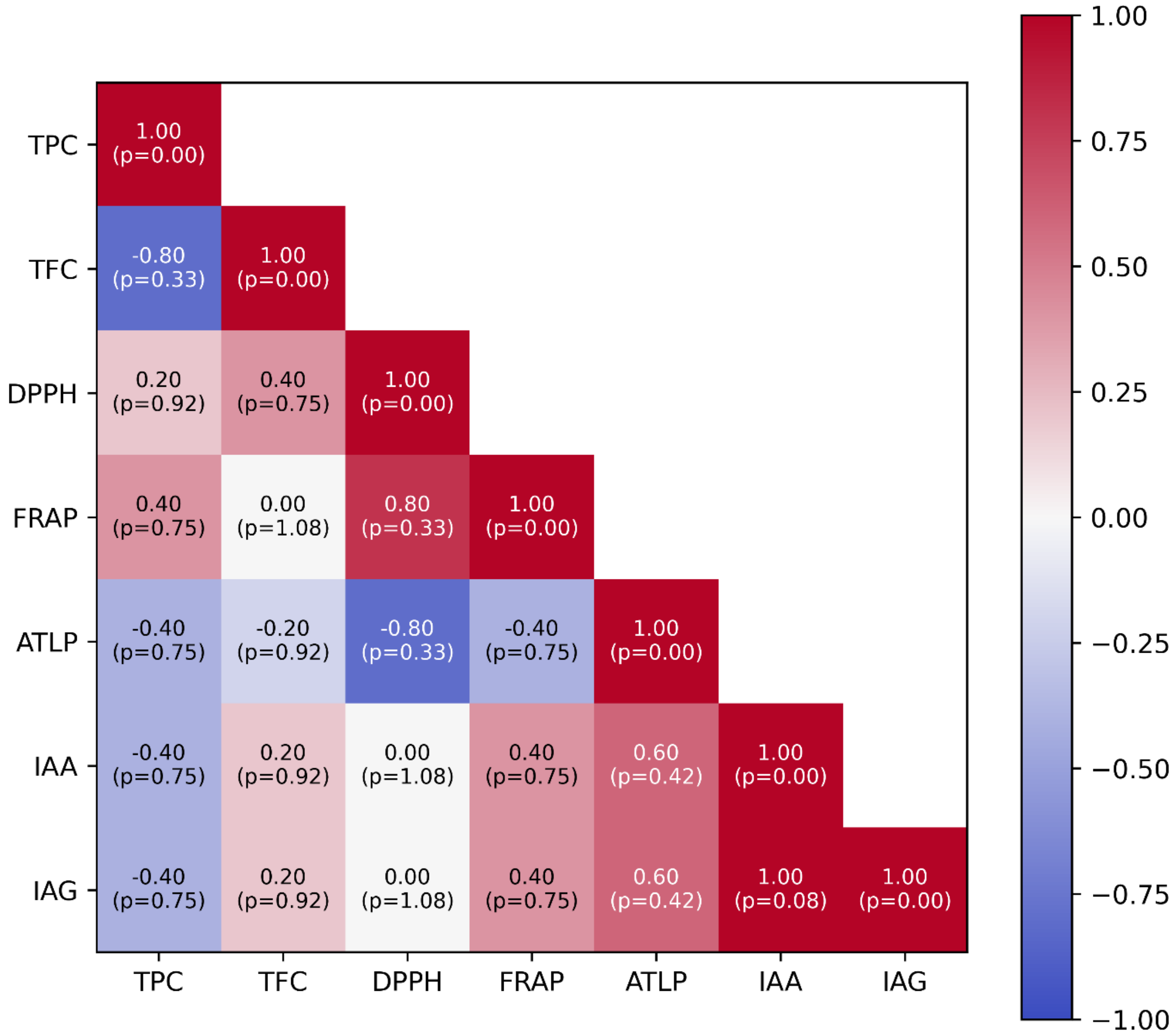

Figure S1. Spearman's rank correlation analysis of phytochemical contents and biological activities.

Note: total phenolic content, TPC; total flavonoid content, TFC and biological activities, including antioxidant assays (DPPH and FRAP), anti-lipid peroxidation activity (ATLP), and inhibitory activity of α-amylase (IAA) and inhibitory activity of α-glucosidase (IAG). Correlation coefficients (ϱ) and corresponding p-values are indicated. Positive and negative correlations are represented by red and blue colors, respectively.

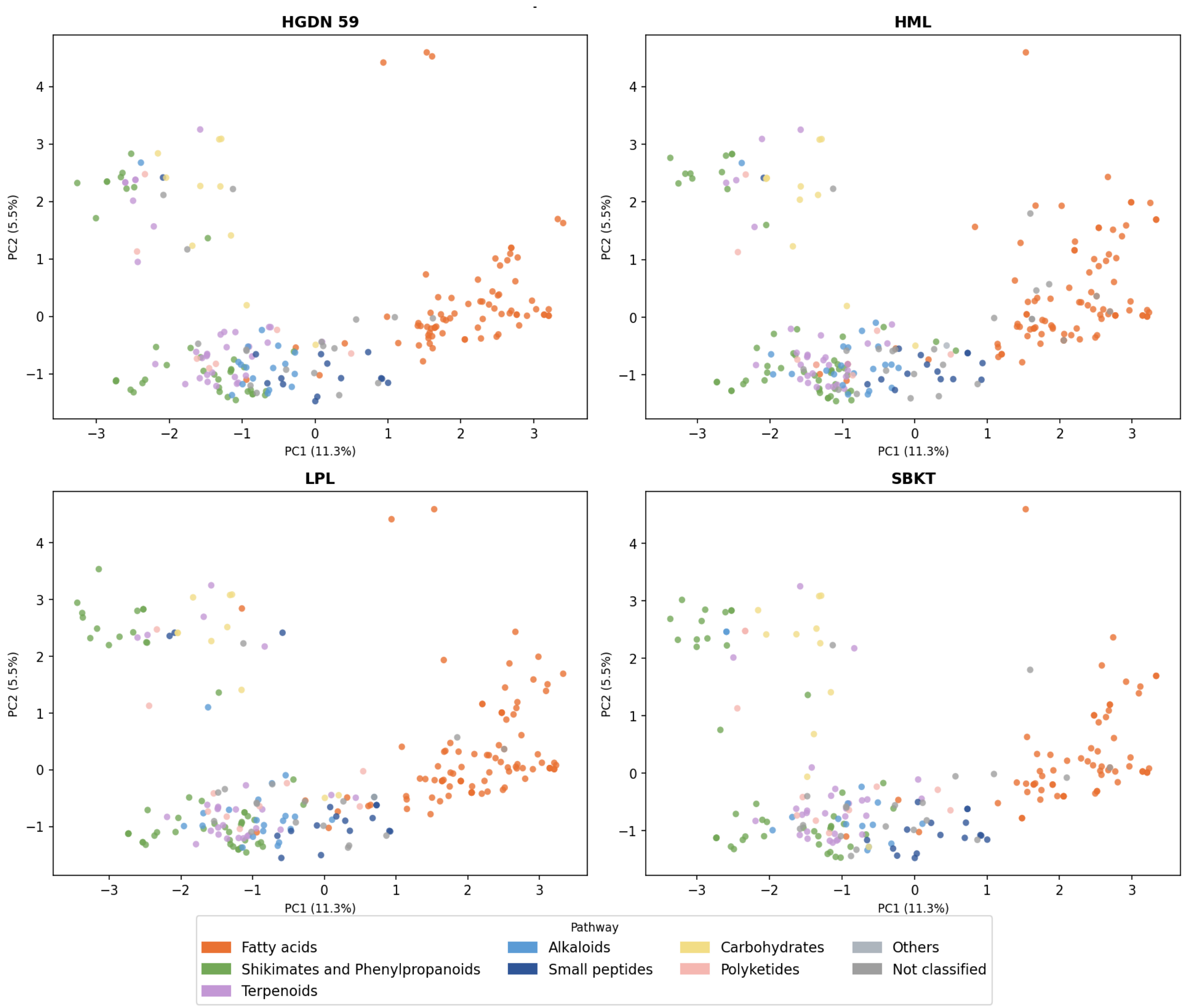

Figure S3. The PCA plots of the chemical space of the compositions across HGDN 59, HML, LPL, and SBKT cultivars. Each plot represents a single cultivar, and the compounds are highlighted based on their pathways.

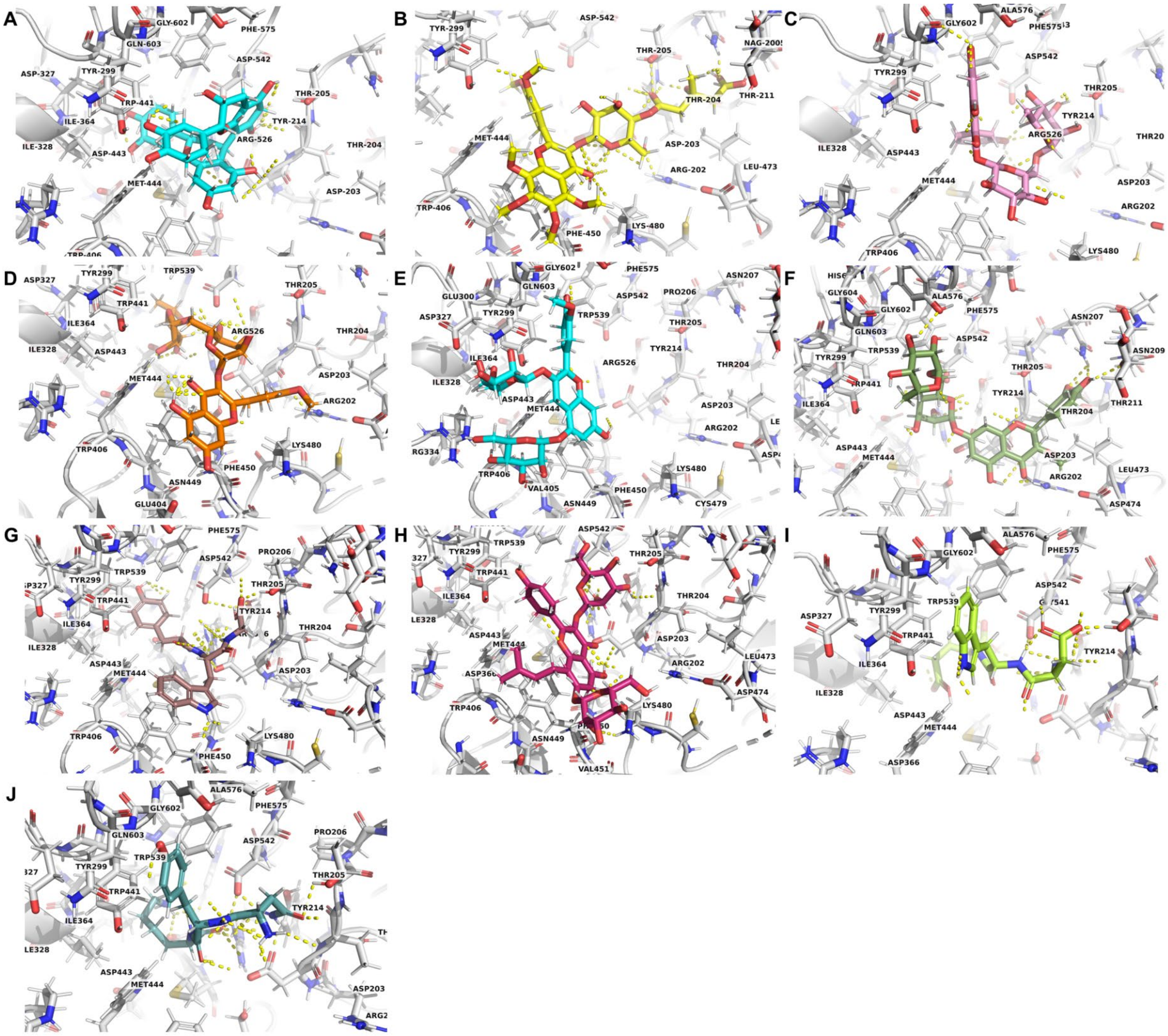

Figure S4. Three-dimensional binding poses of compounds identified from HGDN 59 (negative mode), HML (negative and positive modes), LPL (negative and positive modes), and SBKT (negative and positive modes) cultivars in the binding pocket of α-glucosidase. Binding conformations are shown for A. Procyanidin B2 (cyan), B. Natsudaidain 3-(4-*O*-3-hydroxy-3-methylglutaroylglucoside) (yellow), C. Rutin (pink), D. 3-*O*-[β-L-rhamnofuranosyl-(1→6)-D-glucopyranoside] (orange), E. Peonidin 3-galactoside-5-glucoside (aqua), F. Quercetin 3,3′-dimethyl ether 7-rutinoside (olive drab), G. Tyr-Trp-Gly (pale violet red), H. Hexandraside E (deep pink), I. Asp-Trp-Glu (green yellow), and J. Asn-Tyr-Lys (teal). All ligands are displayed as sticks, and the α-glucosidase is represented as a grey stick and ribbon.

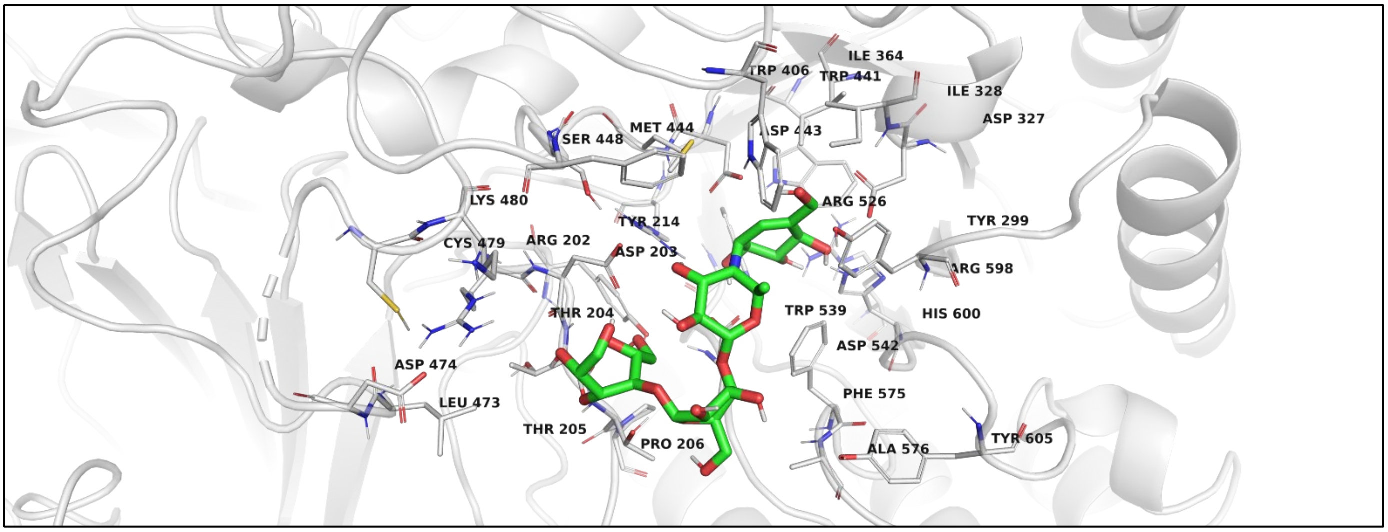

Figure S5. Three-dimensional binding pose of acarbose (green sticks), a known inhibitor of α-glucosidase, within the enzyme's active site (grey ribbon). Key interacting residues are shown as grey sticks and include Arg 202, Asp 203, Thr 204, Thr 205, Pro 206, Tyr 214, Tyr 299, Asp 327, Ile 328, Ile 364, Trp 406, Trp 441, Asp 443, Met 444, Ser 448, Phe 450, Leu 473, Asp 474, Cys 479, Lys 480, Arg 526, Trp 539, Asp 542, Phe 575, Ala 576, Arg 598, His 600, and Tyr 605.

Table S1. Physicochemical properties of other compounds identified in other cultivar extracts targeting α-glucosidase, including molecular formular, MW, LogP, HBD, HBA, TPSA, and structural alert.

No.	Name of compound	Molecular formula	MW	LogP [a]	HBD [b]	HBA [c]	TPSA[d]	Structural alert[e]
			**HGDN 59 (Negative mode)**					
1.	Procyanidin B2	$C_{30}H_{26}O_{12}$	578.526	1.35	10	12	220.76	1
2.	Natsudaidain 3-(4-O-3-hydroxy-3 methylglutaroylglucoside)	$C_{33}H_{40}O_{18}$	724.665	3.60	5	17	248.57	0
			**HML (Negative mode)**					
3.	Rutin	$C_{27}H_{30}O_{16}$	610.521	0.46	10	16	269.43	1
			**HML (Positive mode)**					
4.	Isorhamnetin 3-O-[b-L-rhamnofuranosyl-(1->6)-D-glucopyranoside]	$C_{28}H_{32}O_{16}$	786.689	1.68	12	21	337.58	0
5.	Peonidin 3-galactoside-5-glucoside	$C_{28}H_{33}O_{16}$	625.556	-2.09	10	15	259.75	0
			**LPL (Negative mode)**					
6.	Quercetin 3,3'-dimethyl ether 7-rutinoside	$C_{29}H_{34}O_{16}$	638.575	3.10	8	16	247.43	0
			**LPL (Positive mode)**					
7.	Tyr Trp Gly	$C_{22}H_{24}N_4O_5$	424.457	1.17	6	5	157.54	0
			**SBKT (Positive mode)**					
8.	Hexandraside E	$C_{32}H_{38}O_{16}$	678.64	2.17	10	16	269.43	0
9.	Asp Trp Glu	$C_{20}H_{24}N_4O_8$	448.432	-0.19	17	6	211.91	0
10.	Asn Tyr Lys	$C_{19}H_{29}N_5O_6$	423.47	1.09	7	7	210.86	0

[a]Log *P* values represent the octanol-water partition coefficient.

[b]HBD represents the number of hydrogen bonding donors.

[c]HBA represents the number of hydrogen bonding acceptors.

[d]TPSA represents the polar surface area (Å^2).

[e]Structural alert count reflects the number of toxicophoric substructures identified within each compound, with higher values indicating a greater likelihood of toxicity.
