# Supplementary material for: Comparative Chemical Profiling, Antioxidant Activity, and Antidiabetic Potential of Four Whole-Grain Red Rice Cultivars from Three Southern Border Provinces of Thailand: An In Vitro and In Silico Investigation
Source: Foods. 2026 Apr 28;15(9):1534. doi: 10.3390/foods15091534 (PMC13164330; doi:10.3390/foods15091534)
Supplement: Supplementary file 1 [file foods-15-01534-s001.zip › Figure S3.pdf]

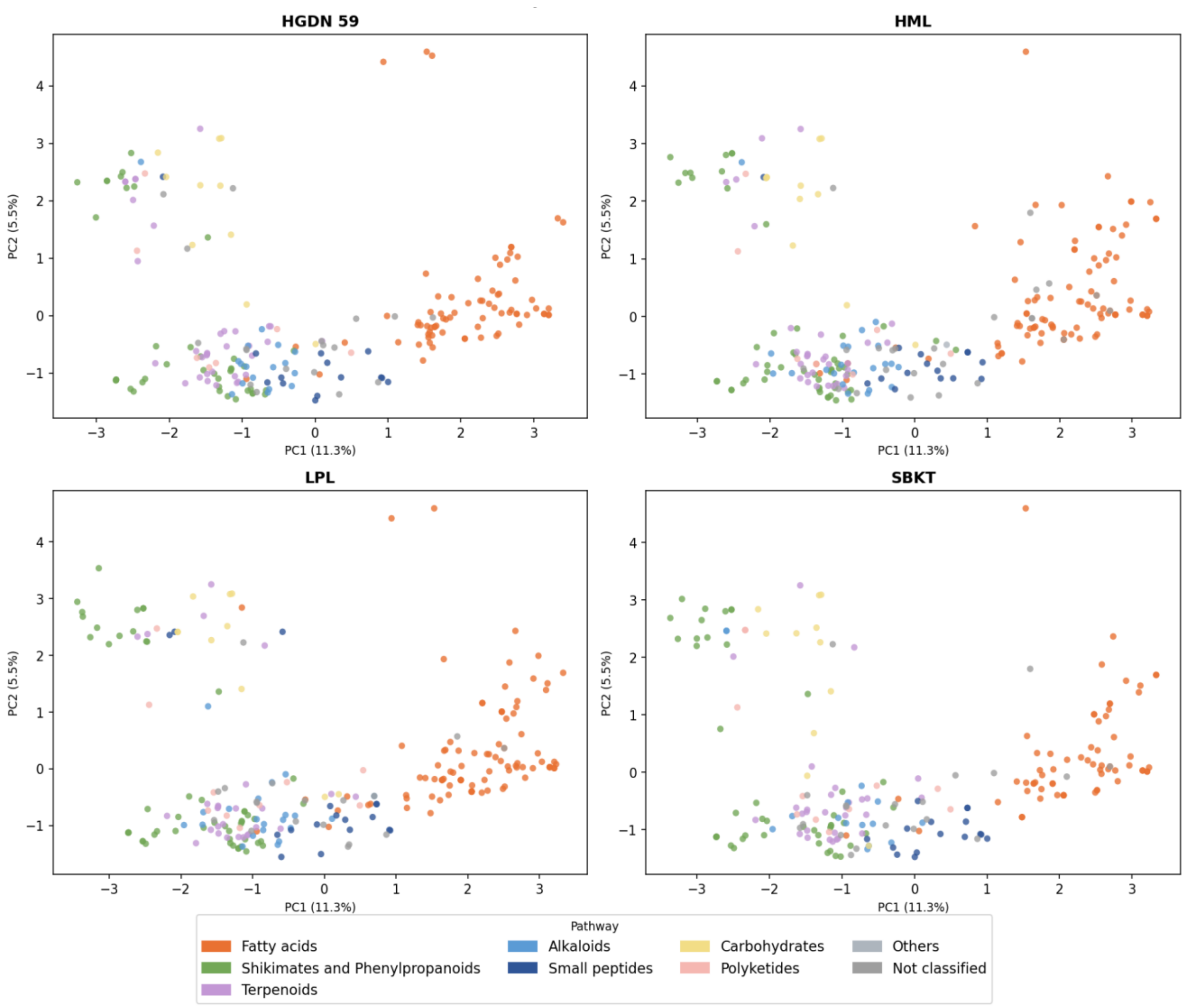

Figure S1. The PCA plots of the chemical space of the ingredients across HGDN 59, HML, LPL, and SBKT cultivars. Each plot represents a single cultivar, and the compounds are highlighted based on their pathways.
