# Supplementary figures and images for: Comparative Chemical Profiling, Antioxidant Activity, and Antidiabetic Potential of Four Whole-Grain Red Rice Cultivars from Three Southern Border Provinces of Thailand: An In Vitro and In Silico Investigation
Source: Foods. 2026 Apr 28;15(9):1534. doi: 10.3390/foods15091534 (PMC13164330; doi:10.3390/foods15091534)

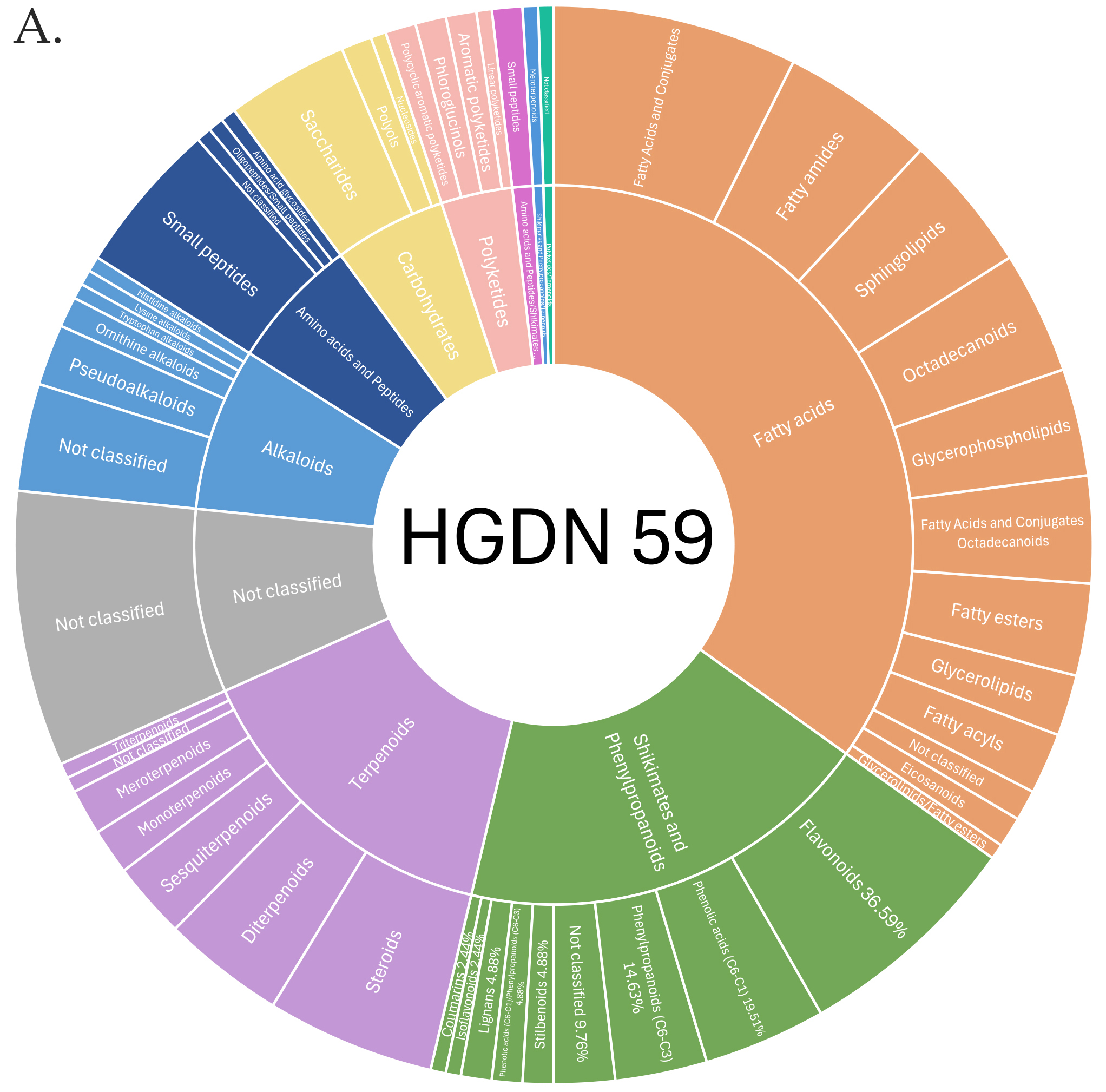

Supplement: Supplementary file 1 [file foods-15-01534-s001.zip › Figure S2/A. HGDN 59.jpg]

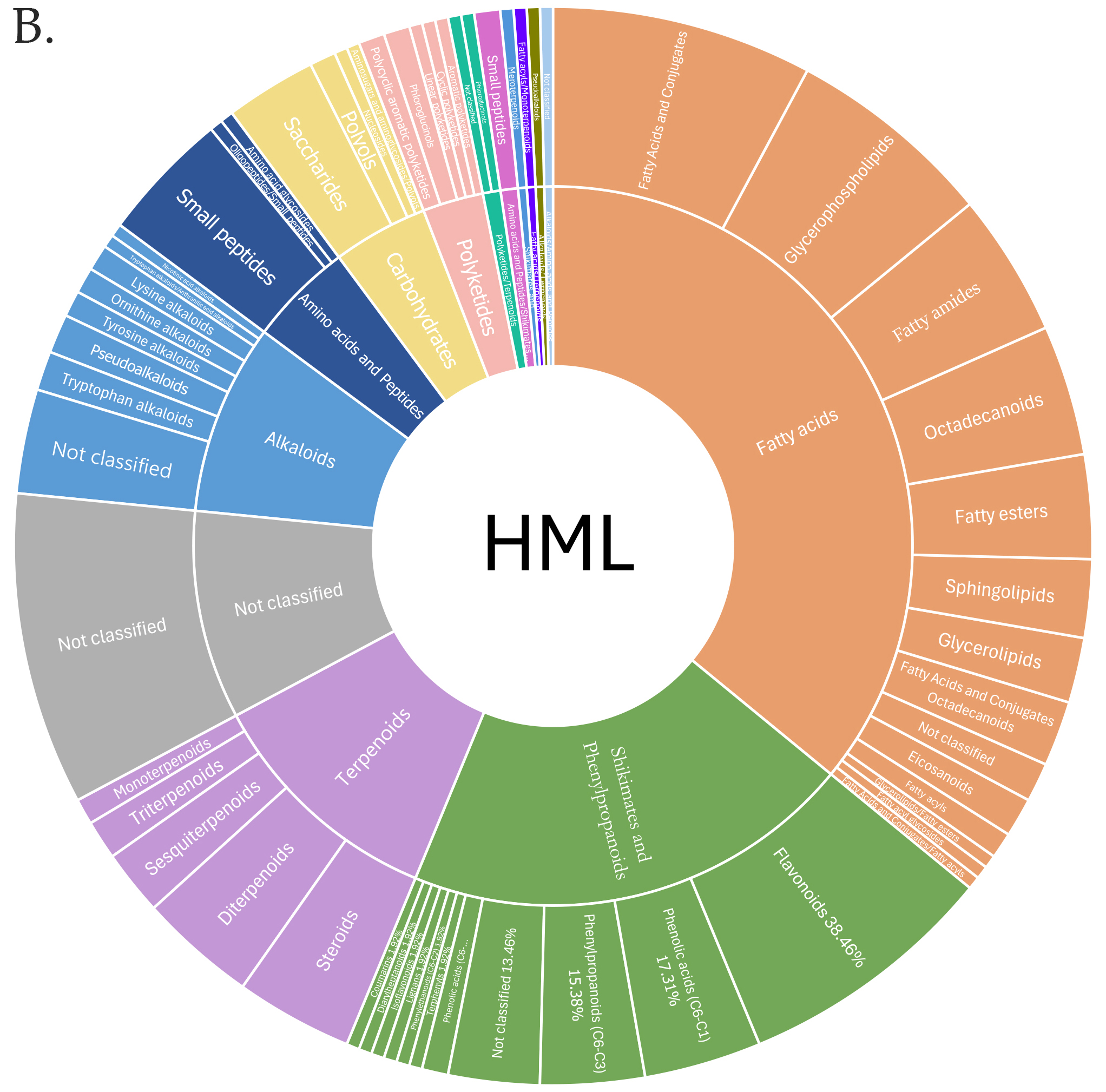

Supplement: Supplementary file 1 [file foods-15-01534-s001.zip › Figure S2/B. HML.jpg]

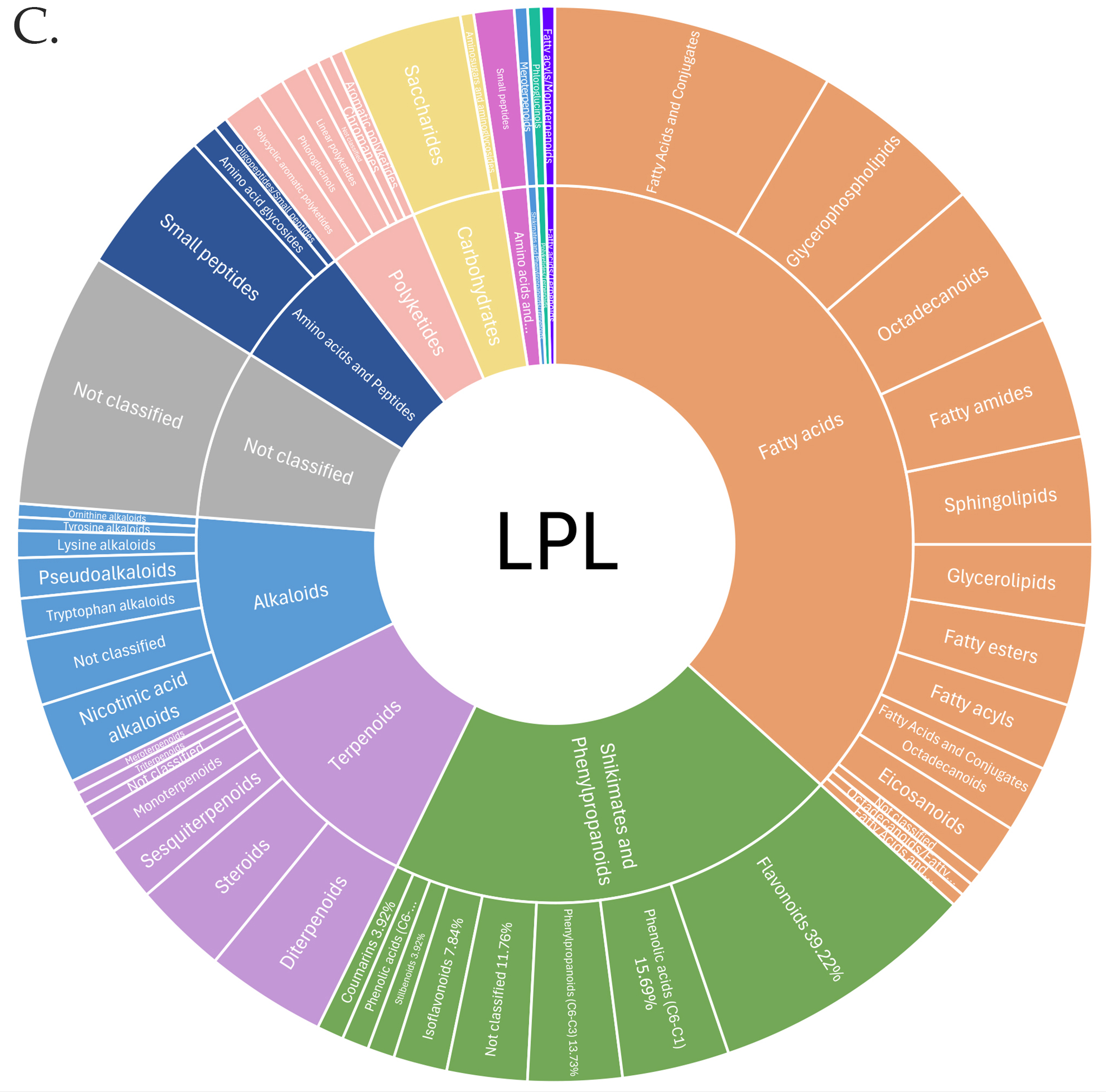

Supplement: Supplementary file 1 [file foods-15-01534-s001.zip › Figure S2/C. LPL.jpg]

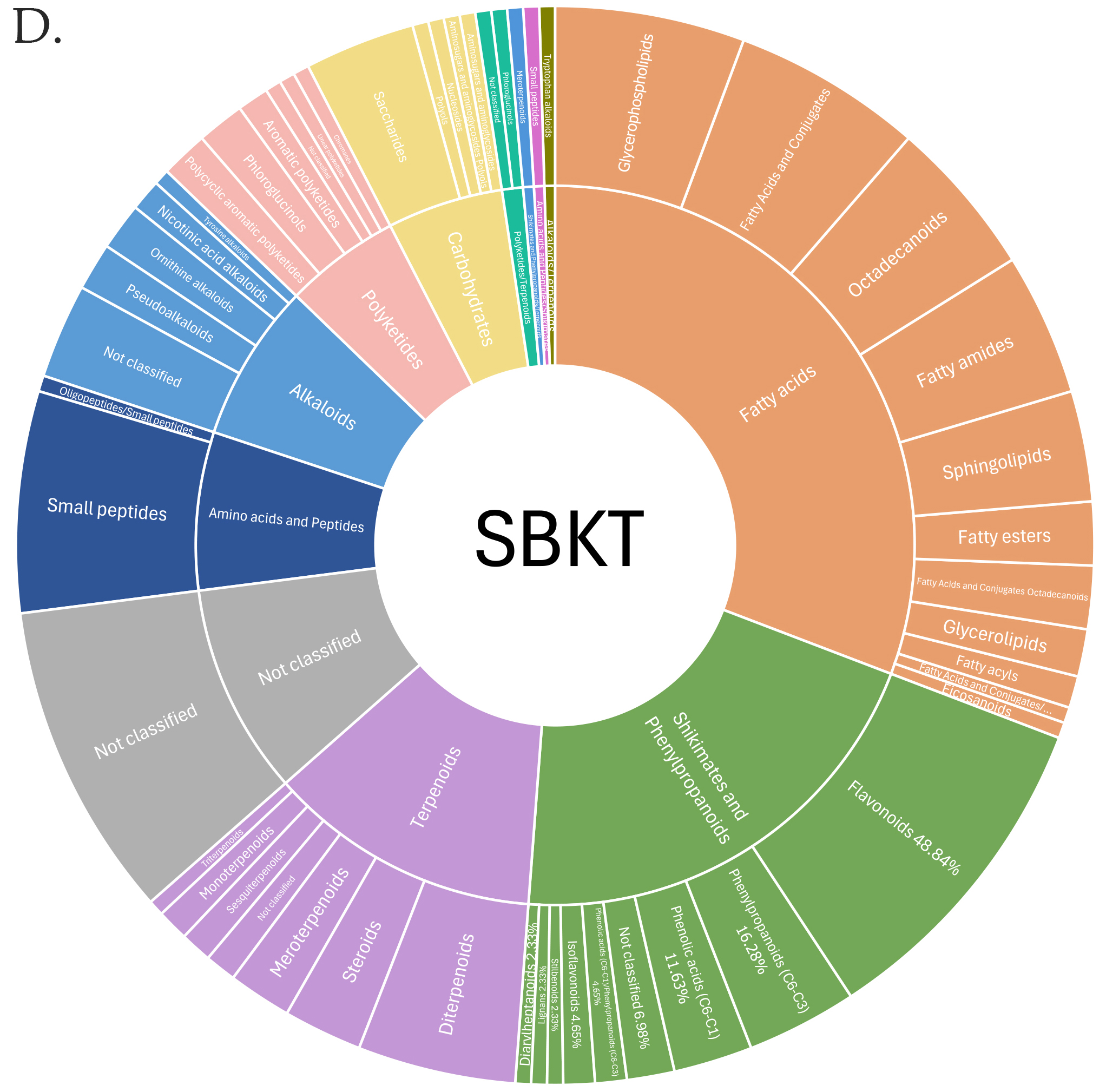

Supplement: Supplementary file 1 [file foods-15-01534-s001.zip › Figure S2/D. SBKT.jpg]

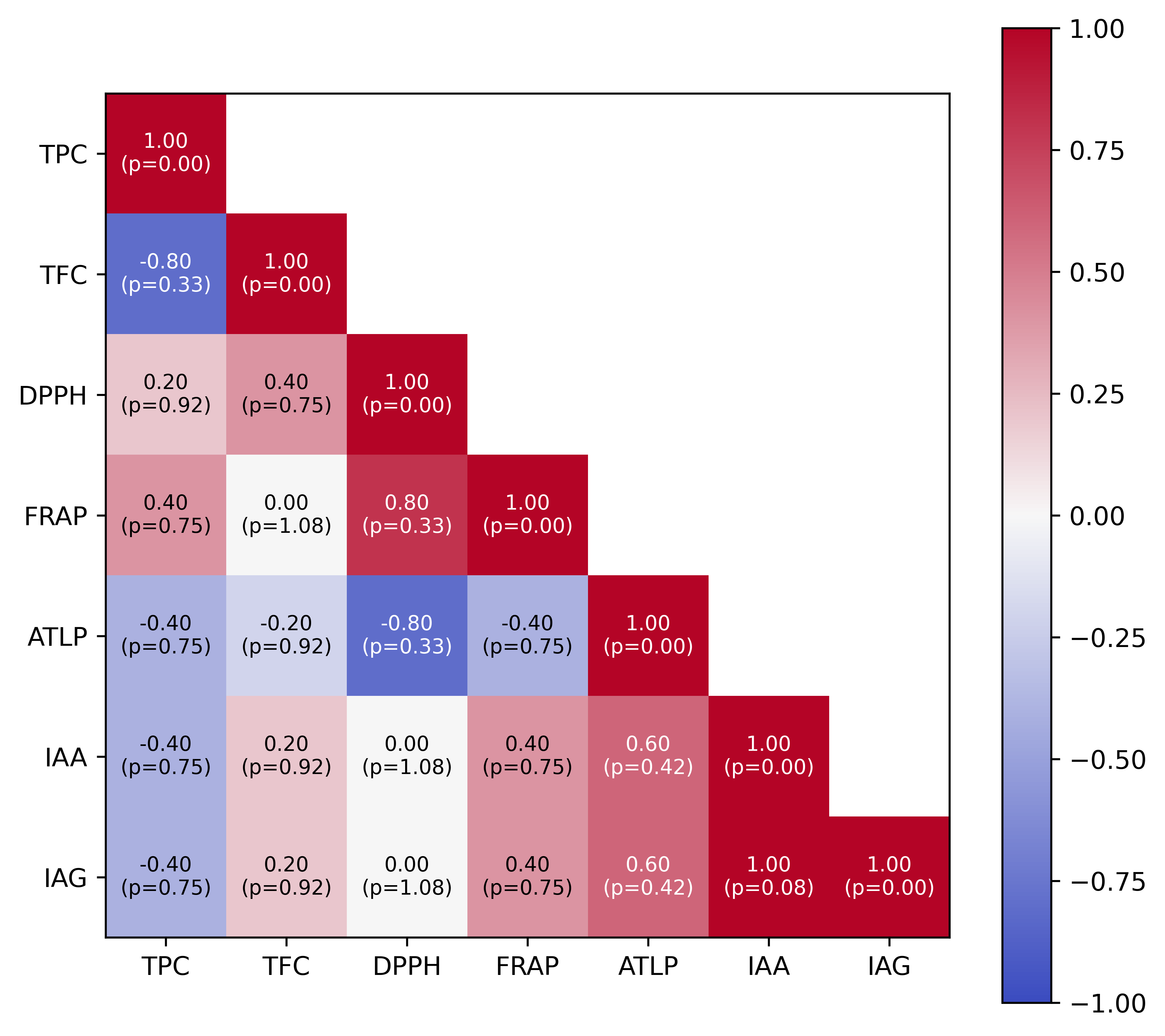

Supplement: Supplementary file 1 [file foods-15-01534-s001.zip › Figure S1.png]

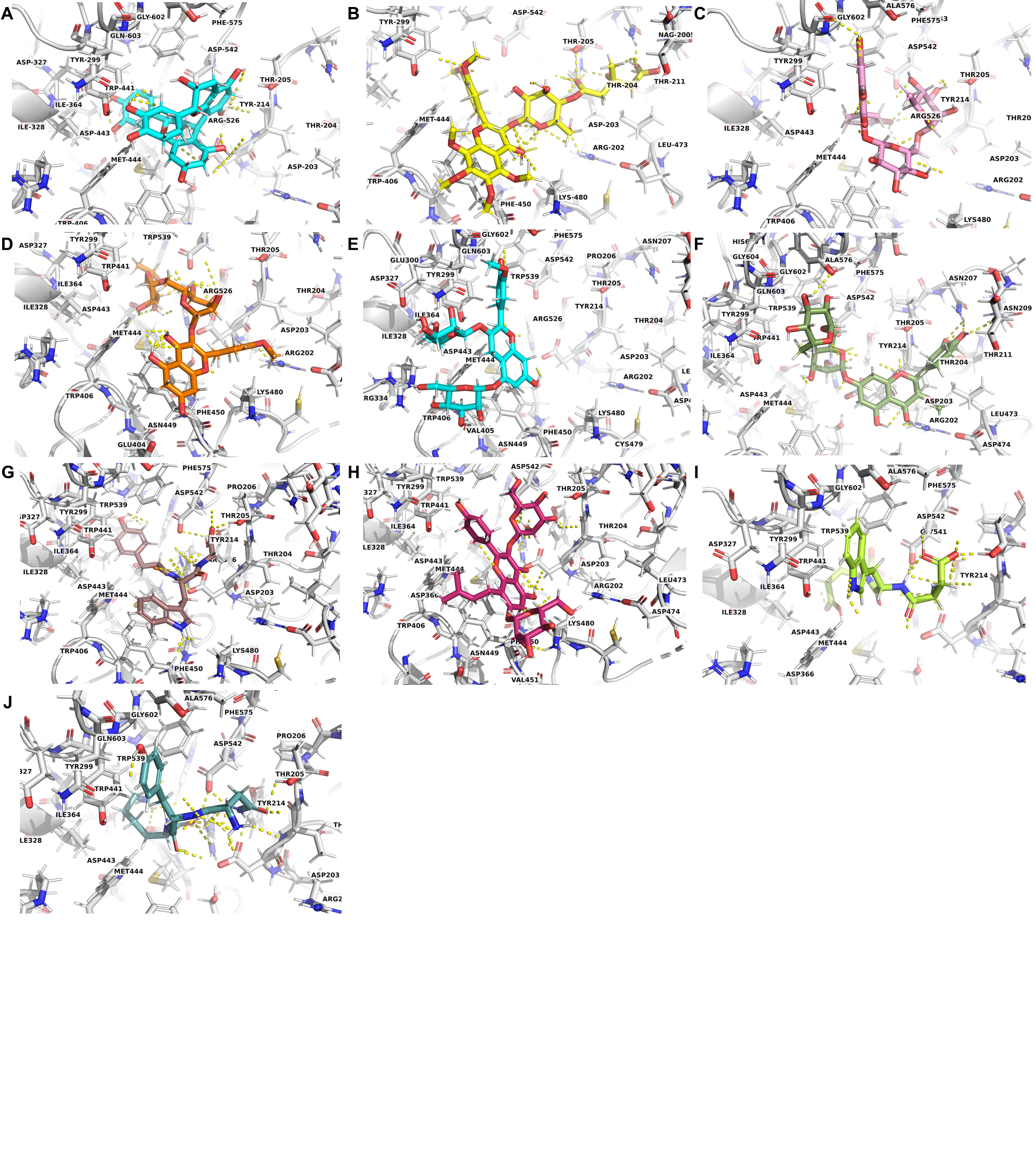

Supplement: Supplementary file 1 [file foods-15-01534-s001.zip › Figure S4.png]

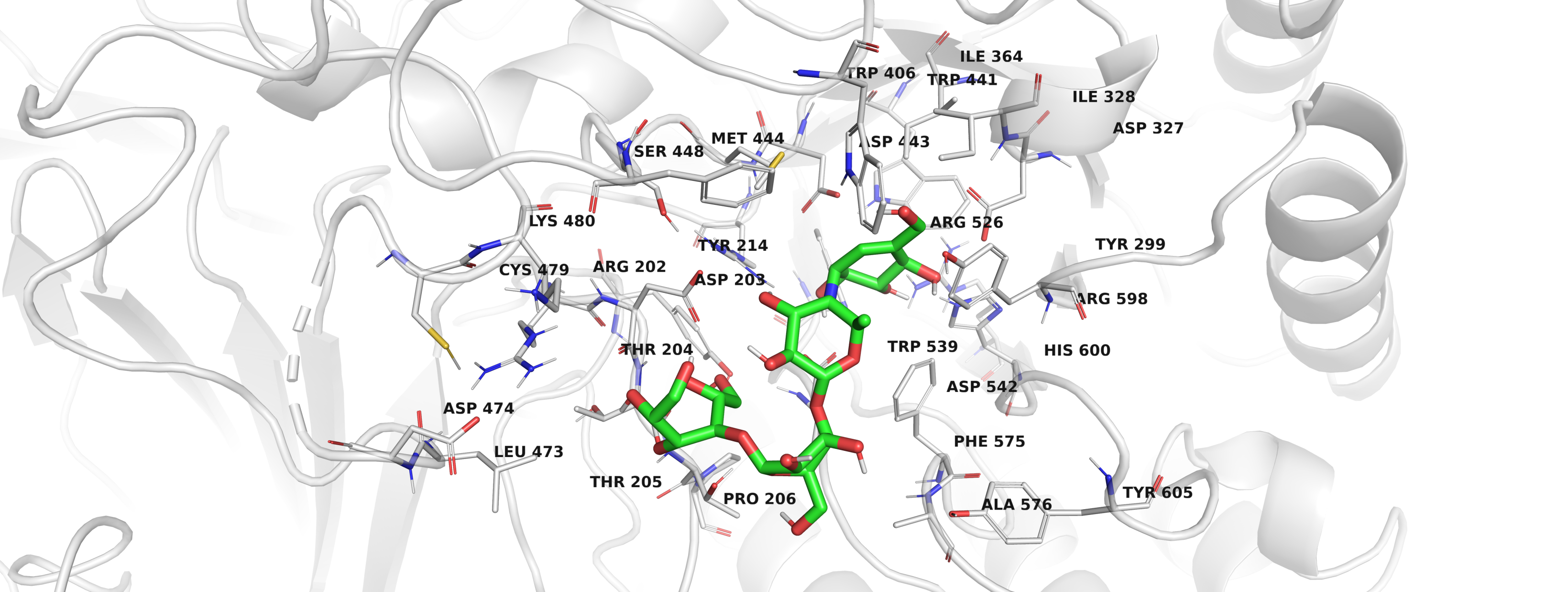

Supplement: Supplementary file 1 [file foods-15-01534-s001.zip › Figure S5.png]
